# Modulation of adenosine A2a receptor oligomerization by receptor activation and PIP_2_ interactions

**DOI:** 10.1016/j.str.2021.06.015

**Published:** 2021-11-04

**Authors:** Wanling Song, Anna L. Duncan, Mark S.P. Sansom

**Affiliations:** 1 Department of Biochemistry, University of Oxford, Oxford OX1 3QU, UK

**Keywords:** GPCR, oligomerization, lipids, molecular dynamics, Markov state models, A2a receptor

## Abstract

GPCRs have been shown to form oligomers, which generate distinctive signaling outcomes. However, the structural nature of the oligomerization process remains uncertain. We have characterized oligomeric configurations of the adenosine A2a receptor (A2aR) by combining large-scale molecular dynamics simulations with Markov state models. These oligomeric structures may also serve as templates for studying oligomerization of other class A GPCRs. Our simulation data revealed that receptor activation results in enhanced oligomerization, more diverse oligomer populations, and a more connected oligomerization network. The active state conformation of the A2aR shifts protein-protein association interfaces to those involving intracellular loop ICL3 and transmembrane helix TM6. Binding of PIP_2_ to A2aR stabilizes protein-protein interactions via PIP_2_-mediated association interfaces. These results indicate that A2aR oligomerization is responsive to the local membrane lipid environment. This, in turn, suggests a modulatory effect on A2aR whereby a given oligomerization profile favors the dynamic formation of specific supramolecular signaling complexes.

## Highlights

• MD simulation and Markov state models are used to study oligomerization of A2aR
• A2aR oligomerization is enhanced by receptor activation and by PIP_2_ interactions
• The population of A2aR oligomers may change in response to environmental cues
• Oligomerization may control combinatory allosteric modulation of GPCR signaling

## Graphical abstract

**Figure:**
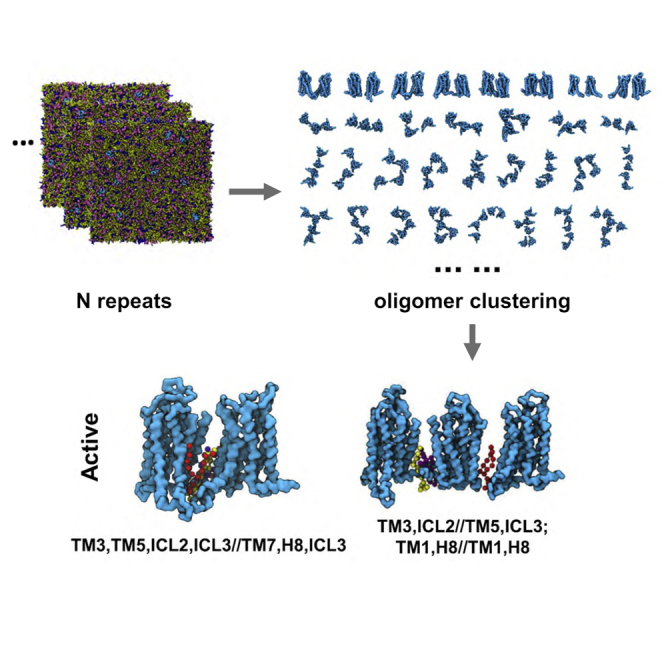

## Teaser

Song et al. use molecular dynamics simulation and Markov state models to show that oligomerization of the adenosine 2a receptor (a class A GPCR) is enhanced by both receptor activation and by PIP_2_ interactions. Oligomerization may influence allosteric modulation of GPCRs.

## Introduction

The lateral organization of cell membranes involves a complex dynamic interplay between the various component proteins and lipids (Jacobson et al., 2019). This is of particular importance in oligomerization and clustering of membrane receptors, which has been explored in detail for, e.g., single-pass receptors (Case et al., 2019). There is an increasing body of evidence for a role for receptor oligomerization in signaling by G protein-coupled receptors (GPCRs) (Dean et al., 2001; Ferre et al., 2014; Simpson et al., 2010; Stauch et al., 2020; Thummer et al., 2005), although the mechanism and exact biological role remain a matter of debate. For example, recent single-molecule imaging techniques have revealed that G proteins recruited by monomers and dimers of the β_2_AR elicited different signaling pathways (Kasai et al., 2020) and the specific effect of the agonist FTY720-P on the S1PR1 was dependent on receptor oligomerization (Patrone et al., 2020). These observations suggest that GPCR oligomers may initiate signaling pathways in addition to those activated by GPCR monomers. Similar to that in monomers, the allosteric modulation of oligomers has been shown to affect the interactions of GPCRs with G proteins and other effectors (Shivnaraine et al., 2016).

Distinctive oligomeric assemblies with various sizes can be formed by GPCRs (Isbilir et al., 2021; Scarselli et al., 2016). The adenosine 2a receptor (A2aR) was found to form higher-order oligomers at the plasma membranes (Glukhova et al., 2017; Vidi et al., 2008a), whereas β_1_ adrenergic receptors formed only monomers and dimers (Calebiro et al., 2013). The kinetics of GPCR oligomerization has been reported to be dynamic (Dijkman et al., 2018) and regulated by receptor density (Calebiro et al., 2013) and by ligand (Möller et al., 2020; Paprocki et al., 2020). The oligomerization landscape is made more complex by diverse oligomeric structures. A couple of GPCR dimer structures have been revealed by X-ray diffraction (Milligan, 2013), and some higher-order oligomeric assemblies have been reported based on studies using single-molecule techniques (Milligan et al., 2019). The biological functions of these various oligomeric structures has been suggested to be specific in terms of their signaling capacities and outcomes (Stoneman et al., 2019).

Molecular dynamics (MD) simulations provide a tool to investigate GPCR oligomeric structures. Rhodopsin was found to favor dimers with interfaces formed by transmembrane (TM) helices TM5/TM5 and TM1,2,8/TM1,2,8 (Periole et al., 2012).The CXCR4 receptor favors dimer interfaces formed by helices TM1/TM5-7 and TM3,4/TM3,4 (Pluhackova et al., 2016). MD simulations have also revealed the impact of membrane lipids on GPCR oligomerization. Increasing the concentration of cholesterol in membranes correlates with enhanced plasticity and flexibility of dimerization of the 5-HT1A receptor (Prasanna et al., 2016) and of the μ-opioid receptor (Meral et al., 2018; Provasi et al., 2015). Likewise, the presence of phosphatidyl serine (PS) in membranes induces specific dimer interfaces of the NTS1 receptor (Gahbauer and Bockmann, 2020). These findings coordinate with the observation that lipids, e.g., cholesterol molecules and some fatty acyl chains, are frequently seen at dimer interfaces in structures of, e.g., P1Y_12_, A2aR, and β_2_AR, and others (Duncan et al., 2020b).

Despite these efforts, our current understanding of GPCR oligomerization remains incomplete, reflecting a sparsity of structural information. Without a full characterization of the quaternary structures of GPCR oligomers, it is difficult to understand how GPCR oligomerization responds to receptor activation and signaling. Here, we provide a comprehensive characterization of oligomerization, quaternary structures, and kinetics of a prototypical class A GPCR, the A2aR, via extensive MD simulation data (∼2.6 ms of CG-MD in total) using *in vivo* mimetic membranes. The A2aR has been demonstrated experimentally to form dimers and higher-order oligomers in native membranes (Vidi et al., 2008a, 2008b). Our simulations reveal that both oligomer quaternary structures and the kinetics of A2aR oligomerization are subject to modulations by the receptor's conformational state and by the lipid interactions of the receptor. Such responsiveness of A2aR oligomerization suggests a combinatory allosteric modulation of GPCR signaling, in which the receptor may respond to the surrounding membrane environment to generate a unique population profile containing monomers and specific dimeric and oligomeric supramolecular complexes. This helps us to understand the structural details of GPCR signaling complexity at a larger scale, presenting new possibilities to manipulate GPCR function.

## Results

### MD simulations sample both association and dissociation of A2aR

**Figure 1:**
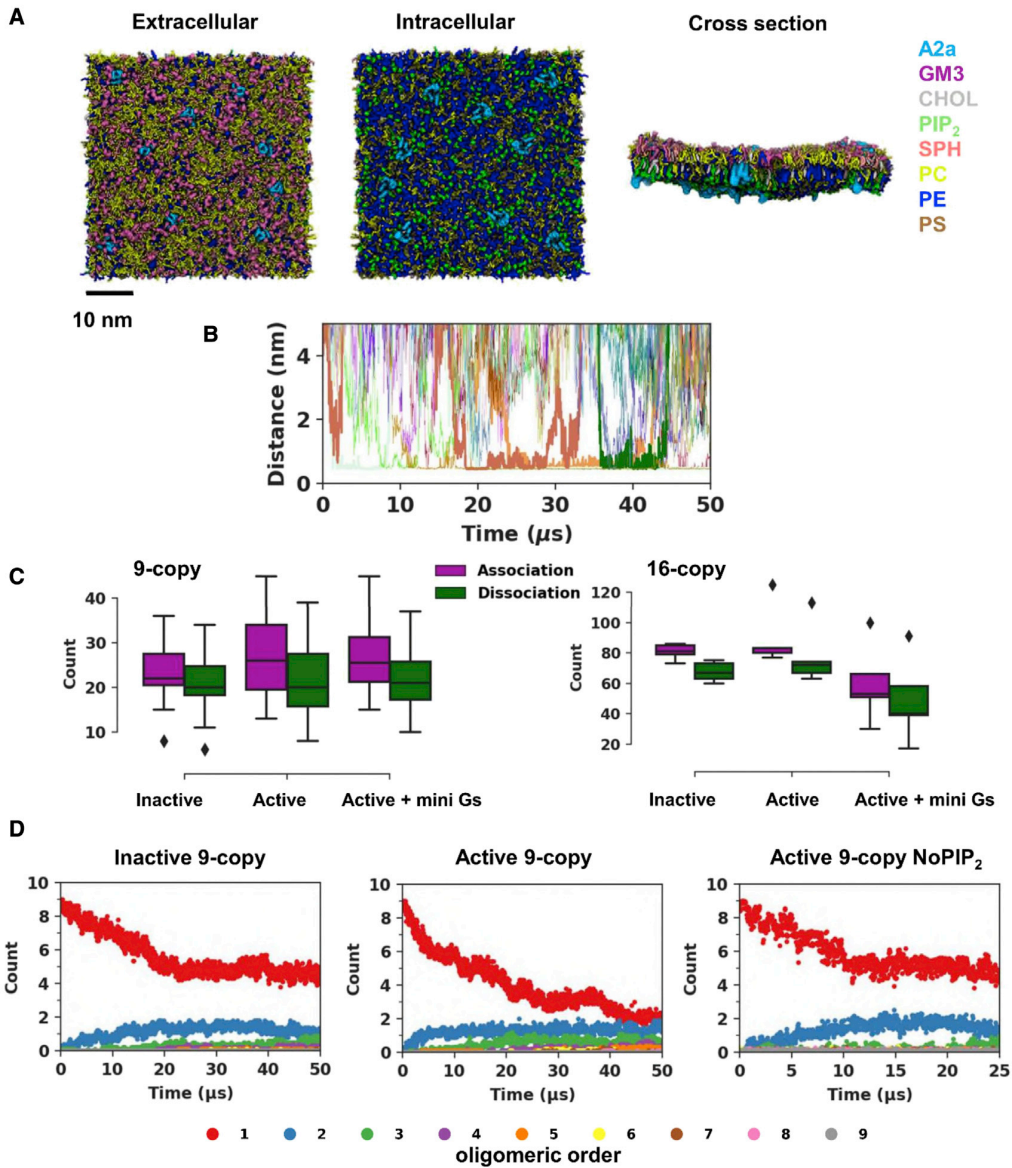
A2aR oligomerization sampled by MD simulations using complex membranes (A) System setup of A2aR oligomerization simulations. The selected number of receptor molecules (pale blue) were randomly inserted into a mixed lipid membrane of area 45 × 45 nm^2^. Views of the systems from the extracellular and intracellular surfaces and in the cross-section are shown, with the lipid species present color coded. Details of the different simulation systems setups are listed in Table 1. (B) The time evolution of minimum distance between pairs of receptors (from a simulations trajectory in the inactive state nine-copy simulation ensemble), illustrating that both association and dissociation events occur within the timescale simulated (selected events are highlighted by bold traces). (C) The number of association (defined as the smoothed minimum distance of a pair of proteins coming closer than 0.75 nm) and dissociation (defined as the smoothed distance of a pair of proteins separating to further than 0.75 nm) events sampled in each trajectory for each of the 9-copy and 16-copy system ensembles. (D) The associations and dissociations have led to a dynamic equilibrium as illustrated by the time course of oligomer formation for the inactive state nine-copy, active state nine-copy, and active state nine-copy NoPIP_2_ simulations. Data averages from all trajectories in the same simulation system were plotted to illustrate the ensemble trend. See Figure S2 for a full list.

We based initial simulations on large-scale membrane systems using a mixture of lipids to form an *in vivo* mimetic model of the plasma membrane environment within which multiple copies of the receptor can freely move, enabling both association and dissociation events to occur. We have focused on oligomerization of the A2aR as this prototypical class A GPCR receptor has been demonstrated experimentally to form dimers and higher-order oligomers (Vidi et al., 2008a, 2008b). We placed 9 copies (positioned and oriented randomly in membrane plane relative to one another) of the A2aR in a 45 × 45 nm^2^ membrane with 10 lipid species present (Figure 1A) to simulate the oligomerization process (i.e., the 9-copy systems). To study how the oligomerization may change in response to receptor activation, we generated three ensembles of simulations for the 9-copy systems, one each for the receptor in the inactive state (PDB: 3EML) (Jaakola et al., 2008), the active state (PDB: 5G53 receptor only), and the active state in complex with a mini Gs protein (PDB: 5G53 receptor and mini Gs) (Carpenter et al., 2016). The simulation systems were modeled using the MARTINI coarse-grained force field (Marrink et al., 2007; Monticelli et al., 2008), including an elastic network to retain the receptor in a given conformation throughout the simulation, thus de-coupling the oligomerization process from receptor conformational changes. We also performed simulations of the three conformational states with a higher protein density (∼4% by area) by including of 16 copies of the receptor in a membrane of the same dimensions (termed the 16-copy), as a number of studies (e.g., Walsh et al., 2018) have shown that altering receptor density can alter GPCR oligomerization. Given recent studies, both computational and experimental (Huang et al., 2020; Song et al., 2019; Yen et al., 2018), demonstrating the interaction of phosphatidylinositol 4,5-bisphosphate (PIP_2_) with class A GPCRs, we also performed simulations with setups identical to the 9-copy system but with a lipid bilayer devoid of PIP_2_ (9-copy NoPIP_2_) to see whether binding of PIP_2_ could modulate GPCRs via altering oligomerization.

Overall, 60 MD trajectories with a cumulative simulation time of more than 2.6 ms were generated (Table 1). The time evolution of the minimum distance between pairs of receptors demonstrated that multiple association and dissociation events occur within the timescales simulated, and protein-protein interactions of different durations were sampled (Figures 1B and S1). On average, each trajectory sampled over 20 association or dissociation events in the 9-copy systems and over 60 in the 16-copy systems (Figure 1C). The total number of association events in the 16-copy systems was approximately (16/9)^2^ times higher than in the 9-copy system, indicating a second-order reaction. The ensemble data of the number of monomers, dimers, and higher-order oligomers suggested that all nine simulation systems approached an equilibrium toward the end of simulations, the characteristics of which were dependent on the membrane environment and the receptor conformational state (Figures 1D and S2). For example, comparing the 9-copy simulations of the inactive versus active states, it can be seen that, after 25 to 30 μs, the number of monomers drops to ∼5 for the inactive state compared with 2 or 3 for the active state. The effect of omitting PIP_2_ from the simulation of the active state of the receptor decreases the tendency of the receptor to oligomerize such that the number of monomers remained at ∼5. The number of monomers in the simulation of active + mini Gs state without PIP_2_ remained at a similar level as in that with PIP_2_. This is likely to be because the protein-protein association in the simulations of active + mini Gs state was to some extent driven by direct interactions between mini Gs and between mini Gs and A2aR (as discussed below), and so the absence of PIP_2_ had a smaller effect on oligomerization.

**Table 1:** Overview of simulations performed

System	A2aR state	No. of copies of protein	Duration (μs) × repeats	Lipid bilayer composition
9-Copy	inactive	9	50 × 10	*Extracellular side:* POPC (20%), DOPC (20%), POPE (5%), DOPE (5%), Sph (15%), GM3 (10%), CHOL (25%) *Intracellular side:* POPC (5%), DOPC (5%), POPE (20%), DOPE (20%), POPS (8%), DOPS (7%), PIP_2_ (10%), CHOL (25%)
	active		50 × 10	
	active + mini Gs		50 × 10	
16-Copy	inactive	16	50 × 5	
	active		50 × 5	
	active + mini Gs		50 × 5	
9-Copy NoPIP_2_	inactive	9	25 × 5	*Extracellular side:* POPC (20%), DOPC (20%), POPE (5%), DOPE (5%), Sph (15%), GM3 (10%), CHOL (25%) *Intracellular side:* POPC (5%), DOPC (5%), POPE (25%), DOPE (25%), POPS (8%), DOPS (7%), CHOL (25%)
	active		25 × 5	
	active + mini Gs		25 × 5	

All simulation boxes were 45 × 45 × 25 nm^3^. Tails: PO: C16:0/18:1 tails. DO: C16:1, C18:1 tails. PIP_2_ molecules had PO tails.

### Oligomerization profiles depend on the conformational state of the receptor

**Figure 2:**
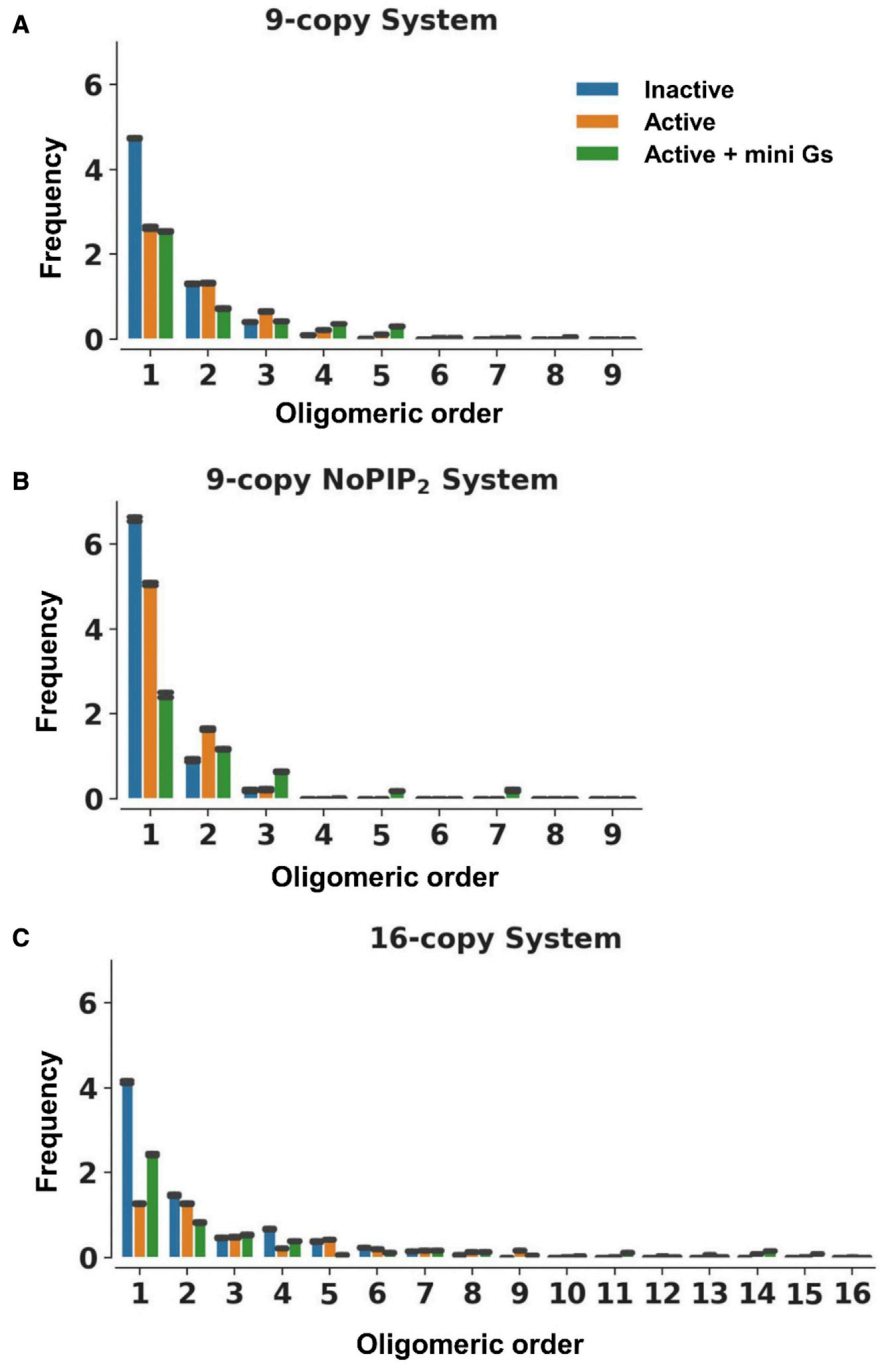
Oligomer distribution The oligomer distributions are shown for the final 20 μs in the 9-copy simulation (A), for the final 10 μs in the 9-copy NoPIP_2_ simulation (B) and for the final 20 μs in 16-copy simulations (C), estimated by counting the number of oligomers in the systems. The error bars (black) denote standard deviations along the trajectory time course.

Determining the distribution of oligomers within each simulation system showed that the active state favors oligomerization (note: we use “oligomerization” to refer to both dimerization and higher-order oligomerization) compared with the inactive state. Thus, for the active state, about 66% of the receptors were oligomeric compared with 40% for the inactive state in the 9-copy systems (Figure 2). Not surprisingly, comparison of the 9-copy and 16-copy simulations indicates that a higher receptor density in the membrane favors oligomerization. The presence of the mini Gs protein bound to the receptor also shifted oligomerization profiles in favor of higher-order oligomers. Increased frequencies of higher-order oligomers were seen for the mini Gs-coupled state in all three sets of simulations. The correlation of oligomerization with both receptor activation and protein density is in agreement with observations on a number of GPCRs (Tabor et al., 2016; Ward et al., 2015, 2017).

**Figure 3:**
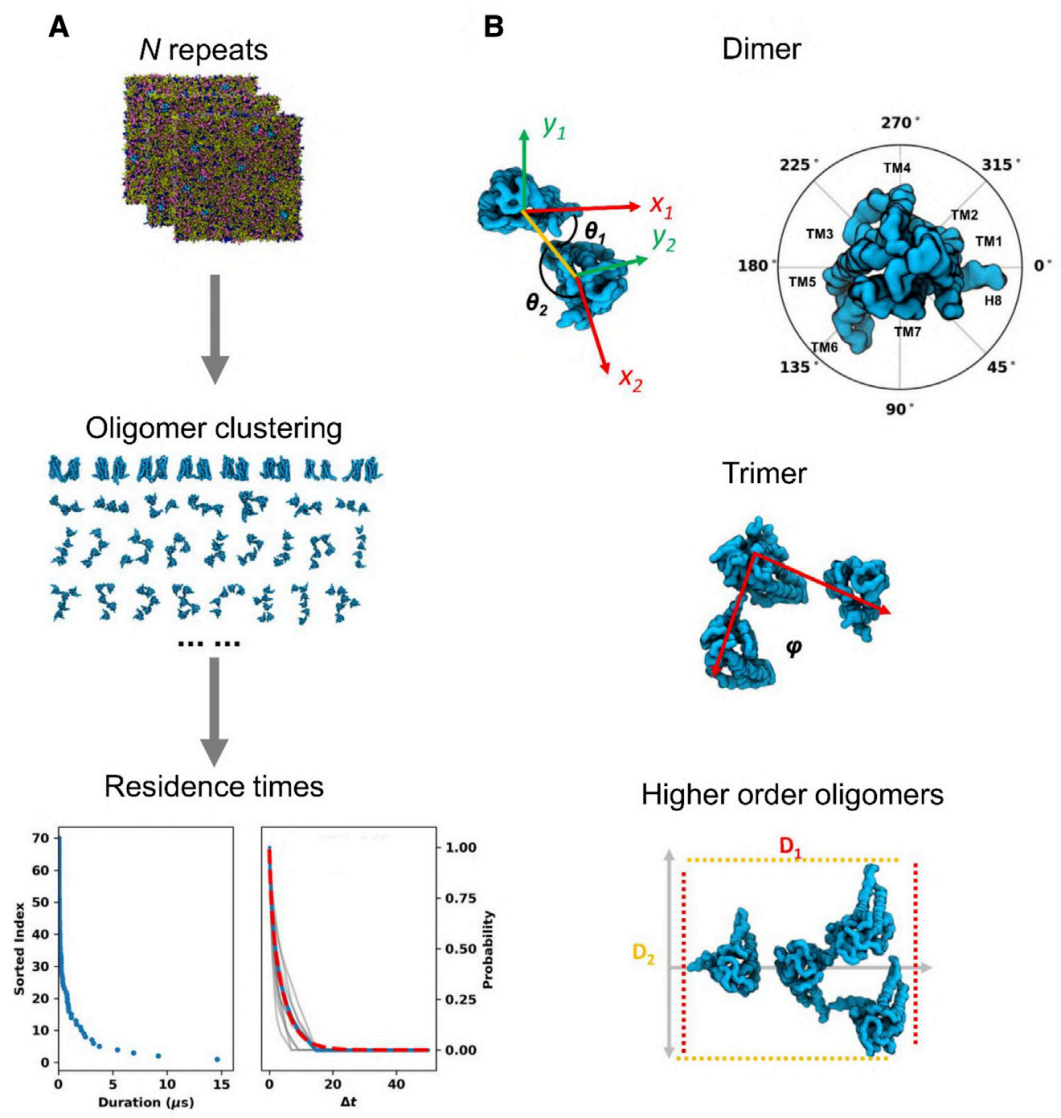
Characterization of oligomer quaternary structures (A) The oligomer quaternary structures from the same simulation set were clustered to identify the various oligomeric configurations. For the calculation of oligomer residence time, durations of each configuration were collected and sorted (blue dots in the left panel). A normalized survival function as a function of Δt (blue dots in the right panel) were modeled based on the sorted durations. A biexponential (red dashed line in the right panel) was used to fit to the survival function to obtain k_off_. To estimate the confidence of the calculated k_off_ values, survival functions based on bootstrapped durations were modeled (gray lines in the right panel) from which standard deviations were calculated. (B) The following metrics were used to describe the oligomer configurations: for dimers, two binding angles (θ_1_, θ_2_), such that each of the angles describes the relative position of the dimer interface to the principal axis of that monomer that is parallel to H8 in a clockwise direction; for trimers, the bending angle ϕ defined by the center of mass of the three monomers; and for tetramers and pentamers, the projected lengths D_1_ and D_2_ on their first and second principal axes.

*Metrics:* to better understand the oligomerization landscape of A2aR, we used two metrics to describe their oligomerization profile: (1) the population of various oligomeric configurations (i.e., oligomer quaternary structures) to measure their relative likelihood, and (2) the residence times of these configurations to provide a measure of their relative stability (and thus the relative strength of the corresponding protomer interactions). We therefore clustered all the oligomer structures using a technique that is invariant to permutations of molecular indexing and calculated the residence time of each oligomer cluster based on their survival functions (see Figure 3A and the STAR Methods).

To assist in the calculation of populations of the different oligomeric configurations, we used the following metrics to describe these quaternary structures (Figure 3B): (1) for dimers, the binding angles (θ_1_, θ_2_) (Gahbauer et al., 2018), which describe the angles defined by the dimer interface and the principal axis of the monomers that is parallel to H8; (2) for trimers, a bending angle ϕ defined by the centers of mass of the three monomers; and (3) for tetramers and pentamers, the projected lengths D_1_ and D_2_ onto their first and second principal axes, respectively. For the 9-copy systems, oligomers of orders lower than pentamers corresponds to 99.97%, 99.93%, and 91.68% of the total populations of the inactive, active, and active + mini Gs states, respectively.

**Figure 4:**
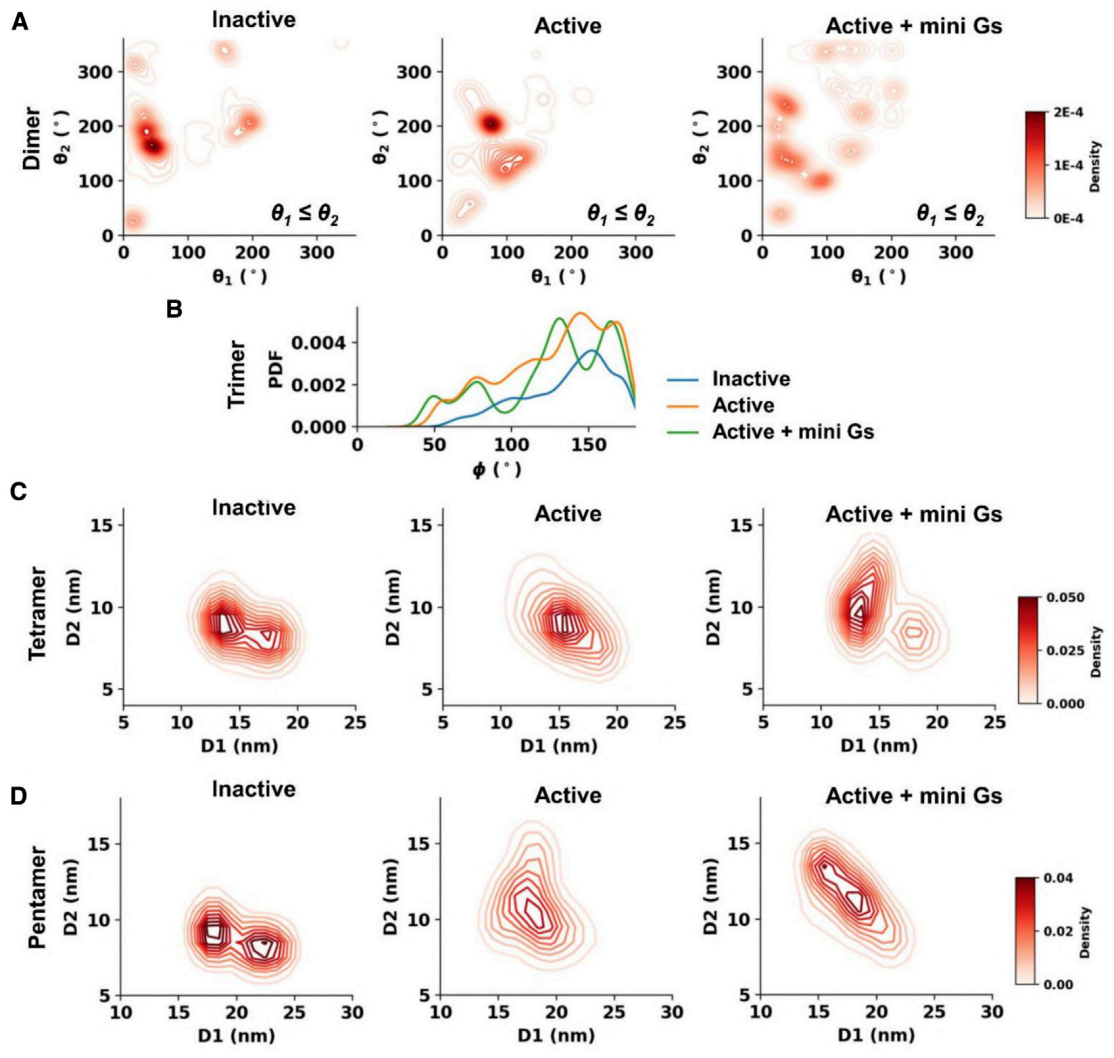
Population profiles of oligomeric configurations in the nine-copy ensemble Distributions of oligomeric configuration metrics defined in Figure 3B are shown in (A) for dimers, (B) for trimers, (C) for tetramers, and (D) for pentamers.

*Dimers:* comparison of the dimeric populations in the 9-copy systems revealed that A2aR dimerization is sensitive to the conformational state of the receptor. Thus, inactive state dimers predominantly showed interfaces: (TM1,H8//TM1,H8) (20°, 20°); (TM3,TM5//TM7,H8) (40°, 170°); and (TM3,TM5,ICL2//TM3,TM5,ICL2) (200°, 200°) (where ICL is the intracellular loop; TMis the transmembrane helix; and A//B indicates an interface between surface A and surface B). When the receptor was in the active state, dimerization around (20°, 20°) and (40°, 170°) became less frequent, whereas the dominant interfaces shifted to those involving ICL3, i.e., interfaces around (TM3,TM5,ICL3//TM7,H8) (70°, 200°) and (TM5,TM6,ICL3//TM5,TM6,ICL3) (130°, 130°) (Figure 4A).

**Figure 5:**
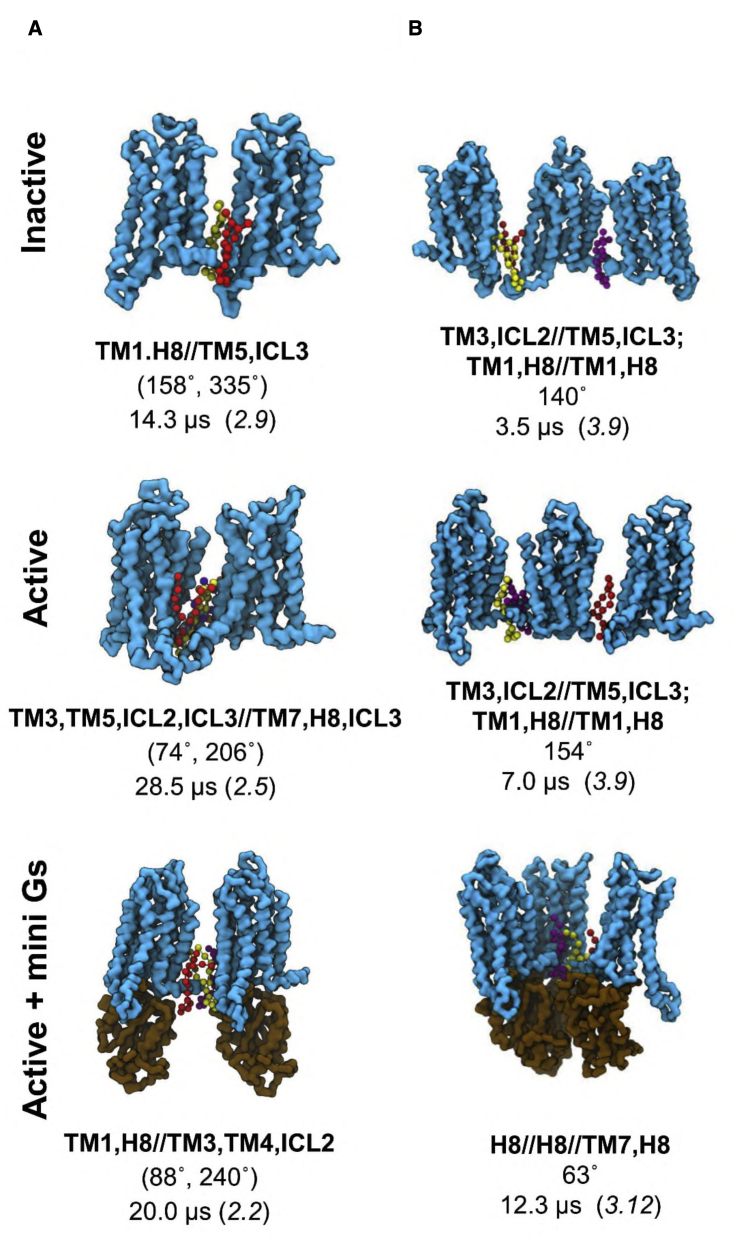
Oligomeric configurations with the longest residence times in the nine-copy ensembles The dimeric (A) and trimeric (B) structures with the longest residence times in each conformational state are shown, with the backbone beads of the receptor in cyan surface and the mini Gs in brown. PIP_2_ molecules at the association interfaces are shown in ball and sticks with different colors. Below each of structures are the association interfaces noted in bold (ICL, intracellular loop; TM, transmembrane helix; A//B, interface between surface A and surface B), along with the average values of the descriptive metrics of oligomer configurations (see text and Figure 3B for details), and the oligomer residence time, which is followed by the cluster ID label in brackets. Also see Figures S3 and S4 and Table S1.

From the dimer residence times, the most stable dimer interface in the inactive state was TM1,H8//TM5,ICL3 (190°, 335°) with a residence time of 14 μs, but only an intermediate population (Figures 4A and 5A; Table S1). This dimer interface is observed in an inactive state of the A2aR crystal structure (PDB: 4EIY). This suggests that residence time, as an indication of oligomer stability, may be a better metric than relative abundance of an oligomeric configuration to predict dimer interfaces in crystallography from simulations. Indeed, the dimer interfaces seen in crystal structures of a couple of other class A GPCRs also ranked highly in terms of residence times for the inactive state. For example, TM3,TM5,ICL2//TM3,TM5,ICL2 (186°, 196°) with a residence time of 10 μs is seen in the crystal structure of CXCR4 (PDB: 3ODU), and TM1,H8//TM1,H8 (30°, 30°) with a residence time of 6 μs is seen in inactive state structures of the β_2_AdR (PDB: 2RH1) and the μ-opioid receptor (PDB: 5C1M; Table S1).

**Figure 6:**
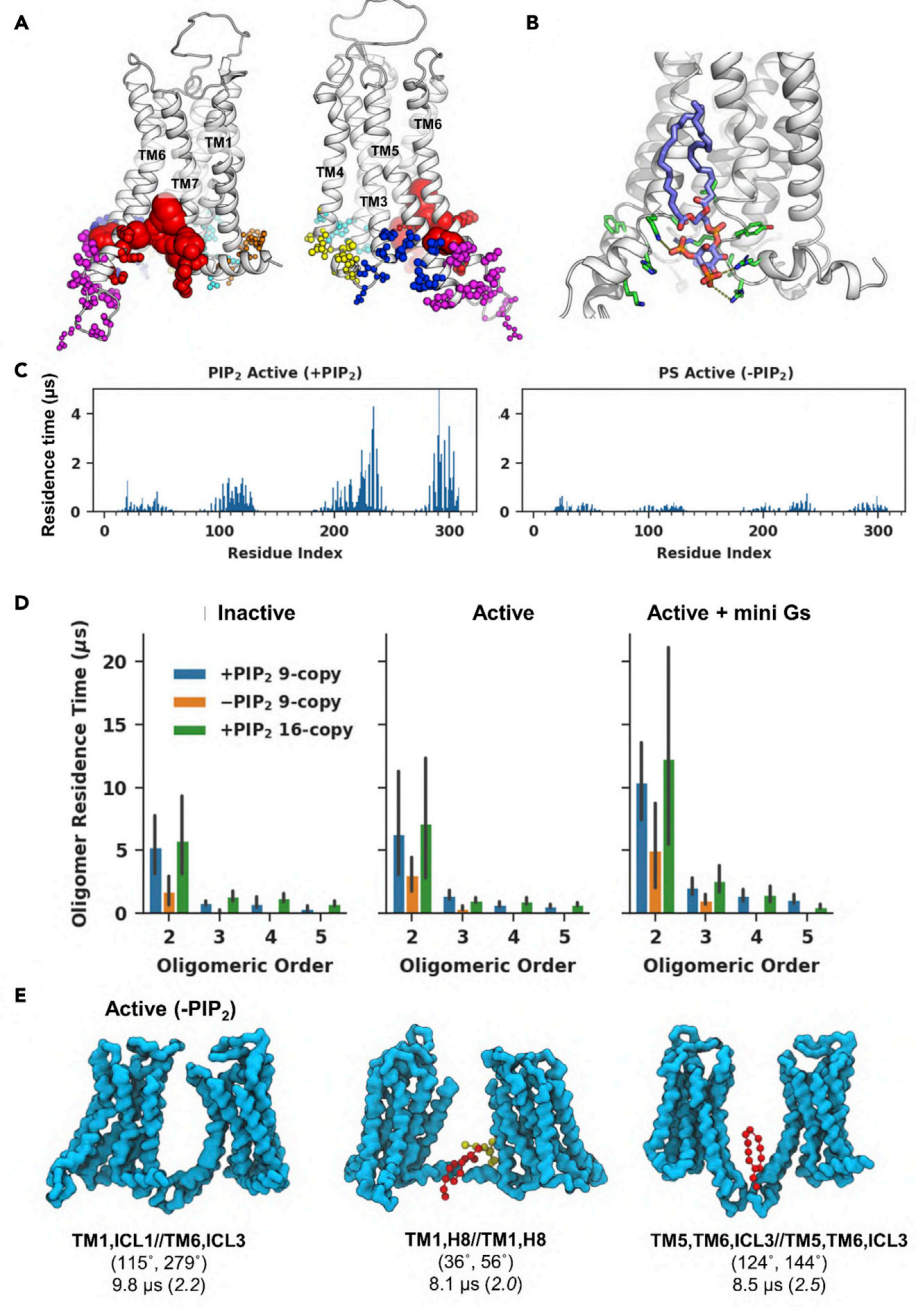
The influence of PIP_2_ on A2aR oligomerization (A) PIP_2_ binding sites on the active state A2aR. The six binding sites calculated using PyLipID (https://doi.org/10.5281/zenodo.4999404) are displayed using different colors. Amino acid residues in each binding site are shown as spheres with radii proportional to their PIP_2_ residence times in the active state nine-copy simulations. (B) A representative PIP_2_ pose bound to the strongest PIP_2_ binding site from the active state simulations (corresponding to the red spheres in A) shown as a back-mapped atomistic structure. Binding site residues are shown as green sticks, and the PIP_2_ molecule as slate-blue/orange/red sticks. Electrostatic interactions between the PIP_2_ head group and protein residues are indicated by yellow dashed lines. (C) Comparison of (left panel) PIP_2_ residence times versus residue number in active state PIP_2_-containing simulations (9-copy) with (right panel) PS residence times in the active state PIP_2_-free simulations (9-copy NoPIP_2_). (D) Comparison of the average residence time as a function of oligomeric order between the three membrane environments (PIP_2_-containing, 9-copy; PIP_2_-free, 9-copy; and PIP_2_-containing, 16 copy) for the three states of the A2aR. Bar heights denote the average residence time of the oligomeric configurations from the same oligomeric order and the error bars show the 95% interval of 1,000 bootstrapped samples. (E) Active state dimer configurations with the three longest residence time from PIP_2_-free simulations. The backbone beads of the receptor are shown in cyan. The PS molecules at the association interfaces are shown in ball and sticks with different colors. Below each of the structures are the association interfaces in bold, the average values of the binding angles, and the oligomer residence time, which is followed by the cluster ID label in brackets. Also see Figure S7.

Stabilities of dimer interfaces in the active state were enhanced (Figure 6D), with a mean residence times of 6.2 μs (range 1–28 μs), compared with 5.3 μs (range 0.8–14 μs) for the inactive state dimers. The most stable dimer in the active state with the interface TM3,TM5,ICL2,ICL3//TM7,H8,ICL3 (74°, 206°) had a residence time of 28 μs (Figure 5A). This resembles a stable inactive interface TM3,TM5//TM7,H8 (41°, 172°) with a residence time of 8 μs (Figure S3) but contained additional inter-protomer interactions at the intracellular side of TM6 and ICL3 relative to its inactive counterpart. This stable active interface also had the largest relative population. Another active highly populated dimer interface, TM5,TM6,ICL3//TM5,TM6,ICL3 (120°, 153°), ranked second in terms of residence times (10 μs). A similar dimer interface can be found in the inactive state at TM3,TM5//TM5,ICL3 (96°, 164°), in which inter-protomer contacts of the intracellular side of TM6 in the active state are replaced by TM5 in the inactive state, reducing the residence time to 2 μs. The active state dimer interface ranked third in residence time (9 μs; TM1,H8//TM1,H8 (35°, 46°); Figure S3), again showing increased stability relative to its inactive counterpart (6 μs).

Binding of the active receptor to the mini Gs strengthened the dimer association as revealed by increased hotspots in the population distribution (Figure 4A). The average residence time of dimers was correspondingly increased to 10 μs (range 3–20 μs; Figure 6D). The enhanced stability resulted from the interactions between the receptor of one protomer and mini Gs of the other in addition to interactions between two receptors. The dimer association hotspots in the mini Gs-coupled state merged those from both the inactive and active states, suggesting that the dimer associations were more promiscuous; however, the stabilities of dimer interfaces, i.e., the ranking of residence times, agreed reasonably well with those in the active state (Table S1), indicating that the association stability was, to a large extent, governed by the activate state of the receptor.

*Trimers and higher-order oligomers:* the residence times of trimers and higher-order oligomers were much lower than for dimers, indicating that the oligomerization at higher orders was dynamically unstable (Figure 6D). These oligomers presented more compact quaternary structures in the active state compared with the inactive state, i.e., more bent trimers with smaller ϕ or more branched/closed higher-order oligomers with similar values of D_1_ and D_2_ (Figures 4B–4D and S4). This shift resulted from the opening at the intracellular side of TM6 and TM5 in the active state that increased the area of the intracellular receptor surface formed by H8-TM6-ICL3 and ICL2-TM5-ICL3. Such a shape made branched/closed quaternary structures more energetically favorable. Dynamic and compact oligomeric structures have been reported for the Ste2 receptor by FRET (Paprocki et al., 2020). The coupling of mini Gs further enhanced this shift to more compact configurations in which some of the signaling partners made contacts with one another. Supramolecular organization of oligomeric GPCRs in complex with oligomeric G proteins have been reported based on single-particle photobleaching experiments (Shivnaraine et al., 2016). The interactions between signaling partners in such supramolecular assemblies were suggested to enable communications between them and hence may provide a structural explanation to the allosteric modulations in GPCR oligomers (Shivnaraine et al., 2016).

### PIP_2_ enhances protein-protein associations and change association interfaces

Inspection of the 9-copy simulation trajectories revealed that the protein-protein associations were mediated by lipid molecules, predominantly by PIP_2_ molecules as shown in Figure 5. The radial distribution of lipids around the receptor (see Figure S5) confirmed that PIP_2_ accumulated around the receptor. We used graph theory and community analysis (see the STAR Methods for details) to characterize the binding sites and binding kinetics. Six PIP_2_ binding sites on A2aR were pinpointed based on the interactions of lipid head group beads (Figure 6A; also see back-mapped atomistic structures in Figure 6B) and the lipid residence times at these binding sites were calculated (Table 2). The strengths of PIP_2_ interactions with A2aR (as measured by residence times) correlated well with the stabilities of dimeric associations they mediated. Thus, for the inactive state, three strong (i.e., long residence time) PIP_2_ binding sites, TM1_H8, TM3_TM5, and TM6_TM7, contributed to the most stable dimer interfaces in this state, i.e. (TM1,H8//TM1,H8) (20°, 20°); (TM3,TM5//TM7,H8) (40°, 170°); and (TM3,TM5,ICL2//TM3,TM5,ICL2) (200°, 200°) (see above). For the active state, the greatest increase in PIP_2_ residence time was seen at site TM6_TM7 (see Figure 6B for an atomistic representation), which went from 2.4 μs in the inactive state to 12 μs in the active state. A similar increase was seen in the PMF calculation of PIP_2_ binding at this TM6-TM7 site in simulations of a single copy of A2aR (Song et al., 2019), suggesting that this increase of PIP_2_ stability is independent of A2aR oligomerization. This PIP_2_ site mediated the most stable dimer interface (TM3,TM5,ICL2,ICL3//TM7,H8,ICL3) of the active state (Figure 5A). Two other PIP_2_ binding sites, TM5_TM6_ICL3 and TM3_TM5, also showed substantial increase of PIP_2_ residence times on going from inactive to active, again correlated with a shift of dimer associations to those involving ICL3 in the active state. In the mini Gs-coupled state, PIP_2_ residence times at all binding sites increased significantly, due to both enhanced protein-protein association and the contribution of the mini Gs to the expanded PIP_2_ binding sites (Song et al., 2019) (Table 2). The overlapping nature of the PIP_2_ binding sites and the protein-protein interfaces, and the strong correlation of PIP_2_ and protein-protein residence times, suggests that PIP_2_ interactions are a key determining factor in driving A2aR oligomerization as observed in the simulations.

**Table 2:** Anionic lipid binding sites in 9-copy simulations

Binding site^a^	TM1_H8			ICL1_TM4			TM3_TM4_ICL2			TM3_TM5			TM5_TM6_ICL3			TM6_TM7		
A2aR state^b^	I	A	AmG	I	A	AmG	I	A	AmG	I	A	AmG	I	A	AmG	I	A	AmG
**PIP**_**2**_**binding sites in PIP**_**2**_**-containing simulations**																		

Residence time (μs)	1.4	1.6	20.2	0.6	1.0	21.3	0.5	0.5	27.1	1.5	3.2	20.4	0.8	4.9	8.1	2.4	12.1	31.3
No. of PIP_2_ molecules	4.4	2.9	5.4	2.7	2.1	3.1	3.0	3.0	3.3	3.8	3.6	5.1	4.2	5.6	5.1	3.9	4.1	3.4

**DOPS binding sites in PIP**_**2**_**-free simulations**																		

Residence time (μs)	0.2	0.7	1.7	0.2	0.17	1.2	0.2	0.2	3.5	0.06	0.09	2.9	0.2	0.2	2.4	0.2	0.5	3.0
No. of DOPS molecules	1.6	1.6	1.6	1.3	1.4	1.6	1.6	1.6	1.7	1.5	1.5	1.6	1.6	1.6	1.4	1.4	1.8	1.7

a The binding sites are illustrated in Figure 6B, to which the color coding used above corresponds. Binding sites were calculated based on the interactions of head group beads.

b I, inactive; A, active; AmG, active + mini Gs.

*NoPIP*_*2*_*simulations:* to further explore the influence of PIP_2_ interaction on A2aR oligomerization, we calculated the oligomerization residence times and lipid interactions in the 9-copy NoPIP_2_ simulations (i.e., the simulation in a bilayer omitting PIP_2_). Regardless of the conformational state of the receptor, the stability of protein-protein associations was significantly reduced for all oligomeric orders (Figure 6C). This reduced stability had led to a lower overall degree of oligomerization compared with the PIP_2_-containing systems (Figure 2). Tetramers or larger oligomers were rarely seen in these simulations. Lipid radial distributions for the NoPIP_2_ simulations (Figure S5) showed a second peak from PS, with a lower height than for PIP_2_ in the PIP_2_-containing simulations. Visual inspection of simulations confirmed that PS molecules were often seen at the protein-protein interfaces (Figure 6E). The distribution of PS interactions with the A2aR was similar to that of PIP_2_ but with much lower residence times, especially at TM6 and TM7 (residue indices 220–240 and 280–310; Figure 6C). Six PS binding sites, similar to those of PIP_2_, were identified, with faster lipid dissociation kinetics than PIP_2_. The PS binding site at TM6_TM7 did not show a prominent increase of lipid residence time in the active state compared with the inactive state, in contrast with the large increase of PIP_2_ residence time at that site (see above). Accordingly, the strongest dimer interface (TM3,TM5,ICL2,ICL3//TM7,H8,ICL3 [74°, 206°]) in the active PIP_2_-containing simulations was rarely sampled in the active NoPIP_2_ simulations (Figure S6). The binding site at TM3_TM5 exhibited the weakest PS interactions among the six binding sites in both inactive and active states of the NoPIP_2_ simulations but showed one of the strongest PIP_2_ interactions (Table 2). This difference correlated with the absence of the stable dimer interface TM3,TM5,ICL2//TM3,TM5,ICL2 (186°, 196°) in the NoPIP_2_ simulations. Analysis of binding site structures and interactions revealed that, while the amino acid compositions were similar for PIP_2_ and PS binding sites, the PIP_2_ molecules showed much longer interactions with the basic residues than did PS molecules (Figure S7). This suggests that the multivalent charged interactions possible for the PIP_2_ head group determine PIP_2_-protein interactions. Taken together, our data show that the PIP_2_ molecules can stabilize A2aR protein-protein associations, and lead to specific association interfaces that are mediated by strong PIP_2_ interactions with the receptor surface.

### Markov state models reveal more dynamic oligomerization network in the active state and in the presence of PIP_2_

To explore the kinetics of A2aR oligomerization in more detail, we constructed Markov state models (MSMs). MSMs can be used to model oligomerization in terms of a network of transitions between oligomeric states, based on information extracted from MD simulations (Husic and Pande, 2018). We used the oligomeric composition of the MD simulation systems, i.e., the number of monomers, dimers and trimers, etc., in the system, for MSM state decomposition to provide a physical description of the system states. An MSM was estimated for each set of simulations using PyEMMA (Scherer et al., 2015), which reweights the transitions such that the equilibrium kinetics and stationary distributions can be recovered. After statistical validation, the MSMs were used to compute equilibrium probabilities, kinetics, and oligomerization networks (see the STAR Methods for details).

**Figure 7:**
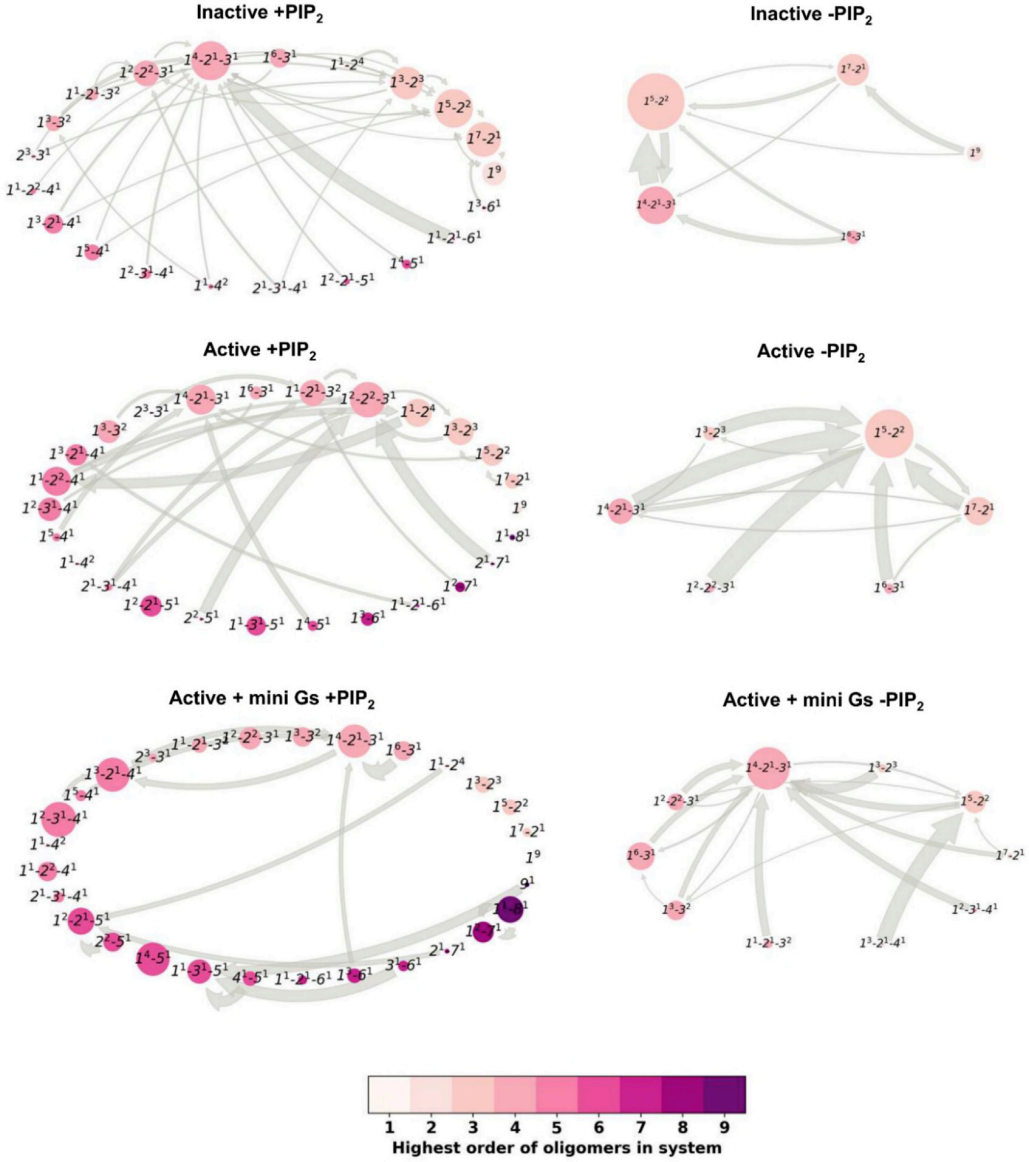
Markov state models of A2aR oligomerization The models calculated the kinetics of the oligomerization by monitoring the evolution of A2aR oligomers in the membrane. The oligomerization states are labeled as *A*^*a*^*-B*^*b*^*-C*^*c*^ in which *A*, *B*, and *C* denote the oligomeric orders present in the membrane, and *a*, *b*, and *c* denote the number of oligomers of the corresponding order. The thickness of the arrows is proportional to the corresponding reaction rate (only reaction rates >50 s^−1^ are shown) and the size of the circles to the equilibrium distributions. Reaction rates were calculated as the reciprocal of the corresponding mean first passage time. To assist visualization, the circles are colored based on the highest oligomer order the representing state contains. MSM trajectories of 10 ms based on the MSM transition probabilities can be found in Figure S8.

The MSMs thus constructed revealed that the inactive state 9-copy systems preferably stayed at the following system states: *1*^*9*^ (i.e., nine monomers), *1*^*7*^*-2*^*1*^ (i.e., seven monomers and one dimer), or *1*^*3*^*-2*^*3*^ (i.e., three monomers and three dimers), with lifetimes over 8 μs. In contrast, for the active state 9-copy systems the predominant system states were *1*^*2*^*-2*^*2*^*-3*^*1*^, *1*^*1*^*-2*^*1*^*-3*^*2*^, *1*^*2*^*-2*^*1*^*-5*^*1*^, or *1*^*1*^*-3*^*1*^*-5*^*1*^, with lifetimes over 8 μs. The active state receptor also saw an increase in lifetime and population of system states containing higher-order oligomers. Accordingly, the lifetime of the monomeric state (*1*^*9*^) decreased to 6 μs in the active state (Table S3). The mini Gs-couple 9-copy systems showed extended lifetimes of 14 μs of *1*^*2*^*-3*^*1*^*-4*^*1*^, *1*^*4*^*-5*^*1*^, and had five system states containing oligomers larger than trimers with lifetimes over 8 μs. The long lifetimes of these two MSM system states suggest that the corresponding oligomeric configurations were favored by the presence of the mini Gs either via forming stable interactions between the receptor and mini Gs or via forming stable interactions among the mini Gs proteins. The lifetime of the fully monomeric system state was further decreased to 5 μs in this mini Gs-coupled state. Reaction rates revealed a more dynamic oligomerization network for the active state receptor, with faster transitions between states (Figure 7). In contrast, for the inactive state, the rates of transition between system states were much smaller with the exception of the transition from *1*^*1*^*-2*^*1*^*-6*^*1*^ to *1*^*4*^*-2*^*1*^*-3*^*1*^. Simulated MSM trajectories using a Monte Carlo algorithm and MSM transition probabilities revealed that the system with active A2aR has more heterogeneous populations of oligomers with more dynamic transitions among higher-order oligomers compared with that with the inactive receptors (Figure S8).

The oligomerization networks in the 9-copy NoPIP_2_ systems were much smaller and simpler due to the absence of larger oligomers (Figure 7). The simpler transition networks have led to more homogeneous oligomer populations comprised of monomers, dimers, and trimers (Figure S8). The residence time of the inactive all-monomer system state was 11 μs, longer than its counterpart in the PIP_2_-containing simulations, indicating a low tendency for oligomerization regardless of the presence of PIP_2_. Similar to the PIP_2_-containing simulations, enhanced oligomerization, i.e., decreased residence time of monomeric system state (5 μs), was seen for the active receptors. In particular, dimers were seen in many of the oligomeric system states. Taken together, the MSMs revealed that both the presence of PIP_2_ in membranes and receptor activations have led to enhanced oligomerization of A2aR with more dynamic transitions and heterogeneous oligomer populations.

## Discussion

### Biological relevance

We have provided a comprehensive characterization of A2aR oligomerization configurations and kinetics by large-scale unbiased CG simulations of systems containing multiple copies of the receptors on multi-microsecond timescales, and subsequent construction of MSMs. One of the key findings from this study is that A2aR oligomerization was enhanced by receptor activation. The conformational changes associated with receptor activation, especially the outward tilt of TM6, shifted the oligomerization interfaces to those involving TM6 and ICL3. Our results are in agreement with the mutagenesis study that showed a critical role of ICL3 in formation of configurations related to the functions of GPCR oligomers (Navarro et al., 2010). Given that outward tilt of TM6 upon activation is a common feature in class A GPCRs, we suggest that the enhanced oligomerization might be an element of a shared mechanism for receptor activation. The enhanced oligomerization by receptor activation was accompanied by a more dynamic oligomerization network with more diverse oligomer populations. Based on our results, it is tempting to speculate that the diverse population of oligomers may serve as a pool that can bind to various signaling partners, either in monomeric or oligomeric forms. We observed that the presence of mini Gs selected a couple of specific oligomeric structures and association interfaces. We also noticed that multiple mini Gs can bind to the more compact association interfaces of, e.g., TM3,TM5,ICL2,ICL3//TM7,H8,ICL2 or TM3,ICL2//TM5,ICL3, while a heterotrimeric G protein may need less compact interfaces, e.g., TM5,TM6,ICL3//TM5,TM6,ICL3 and TM1,H8//TM1,H8. We thus suggest that different signaling partners of A2aR may selectively bind to some of the oligomer structures in the pool. Indeed, different oligomeric structures and association interfaces were reported for, e.g., the Ste2 homodimer (Velazhahan et al., 2020) and M2 tetramers (Shivnaraine et al., 2016), when coupled to their respective G proteins. Our results, therefore, are suggestive of a combinatory modulation of GPCRs whereby the oligomeric population of the receptor could favor certain supra-molecule signaling complexes.

The interactions of membrane lipids, e.g., cholesterols and polyunsaturated fatty acids, have been reported to affect GPCR oligomerization (Guixà-González et al., 2016; Wang et al., 2006), via altering the receptor surface and intercalating between protomers (Gahbauer and Böckmann, 2016). Here, we report a profound effect of PIP_2_, previously shown to interact with a number of class A GPCRs (Huang et al., 2020; Song et al., 2019; Yen et al., 2018), on A2aR oligomerization. We observed a direct correlation between the stability of receptor association interfaces and the strength of PIP_2_ interaction at these locations (e.g., the increase of stability of interface TM3,TM5,ICL2,ICL3//TM7,H8,ICL3 and the increase of PIP_2_ residence time at binding site TM6_TM7). The impact of PIP_2_ on A2aR oligomerization was further confirmed by comparisons with the NoPIP_2_ simulations, in which receptor oligomer residence times were significantly reduced while the dominant PIP_2_-mediated interfaces were absent. Given that our previous simulation studies have shown that the interactions of PIP_2_ are largely conserved across class A GPCRs (Yen et al., 2018), we suggest that the effect of PIP_2_ on GPCR oligomerization may also be shared. Such lipid interactions could be important in determining GPCR oligomerization, e.g., in functionally important nanodomains (Calebiro and Sungkaworn, 2018) with a high local concentration of PIP_2_ (van den Bogaart et al., 2011). It is also possible that the intrinsically disordered C termini of GPCRs (Nguyen et al., 2020) may play a role in interactions with anionic lipids, such as PIP_2_, especially as they contain multiple basic residues (Tovo-Rodrigues et al., 2014).

### Methodological considerations

Simulation studies using the MARTINI force field have provided valuable insights into association of membrane proteins (Chavent et al., 2014; Domanski et al., 2018; Gahbauer and Bockmann, 2020; Meral et al., 2018; Periole et al., 2007, 2012, 2018) and into protein-lipid interactions (Corradi et al., 2018; Duncan et al., 2020a; Hedger and Sansom, 2016). The current MARTINI model has some limitations (Alessandri et al., 2019; Javanainen et al., 2017). In particular the smoothing of free energy landscapes in a coarse-grained forcefield may have resulted in more rapid oligomerization kinetics in this study compared with experimental data. However, in the inactive and active simulations, we do not see the apparently excessive protein-protein interactions reported in some other studies (Gahbauer and Bockmann, 2020; Javanainen et al., 2017). This may reflect the use of more complex lipid bilayer compositions in our simulations, compared with, e.g., commonly used binary composition of PC and cholesterol. Future studies of GPCR oligomerization using MARTINI 3 (Souza et al., 2021) may help to resolve some of these differences.

### Conclusions

**Figure 8:**
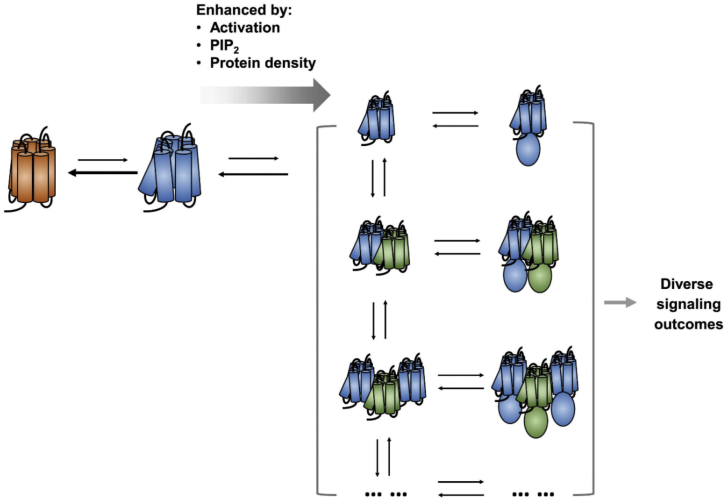
Allosteric modulation and GPCR oligomerization The activated GPCR generates an array of oligomeric assemblies, which couples to signaling partners, generating an array of supramolecular signaling complexes capable of initiating different signaling pathways. This mechanism of combinatory modulation of GPCR is responsive to the lipid bilayer environment and the conformational state of the receptor. Receptor activation, PIP_2_, and/or mini Gs binding, and an increase in receptor density in the bilayer all promote oligomerization.

Reflecting upon our simulation results in the context of experimental studies (Kasai et al., 2020; Navarro et al., 2018; Patrone et al., 2020), we suggest that GPCR oligomerization may provide greater functional flexibility for the receptor signaling array via generating multiple dynamic supramolecular complexes that could initiate different signaling outcomes (Figure 8). From our simulations, we observed enhanced oligomerization with more connected networks in the active state, which resulted in an array of oligomeric configurations capable of coupling to various configurations of oligomeric mini Gs or multiple copies of monomeric mini Gs. In addition, the oligomerization energy landscape was sensitive to the membrane environment. Given that the coupling of G proteins to GPCRs is highly efficient, involving direct collisions with no intermediate (Falkenburger et al., 2010; Hein et al., 2005), the array of functional supramolecular complexes of GPCRs complexed with G protein(s) would be dependent on the array of oligomeric configurations presented by the activated receptor. Our simulations have revealed an additional level of complexity of GPCR allosteric modulation by receptor oligomerization, whereby the receptor can generate a specific array of oligomers according to the environment that would lead to specific signaling complexes and hence functional outcomes. This hypothesis provides a structural explanation for the observation of multiple pharmacological profiles of GPCRs (Ferre et al., 2014), and potentially expands the druggable space to include the protein-protein association interface and allosteric sites corresponding to the protein-lipid interface (see the discussion in Duncan et al., 2020b). Understanding the mechanisms of combinatory modulation of GPCR oligomerization and the role therein of lipid interactions, may present new opportunities for therapeutic targeting of GPCRs.

## STAR★Methods

### Key resources table

**Table:** 

REAGENT or RESOURCE	SOURCE	IDENTIFIER
**Deposited data**		

A2aR inactive state structure	Jaakola et al., 2008	PDB: 3EML
A2aR active state + mini Gs structure	Carpenter et al., 2016	PDB: 5G53
Lipid interaction data	This paper	https://doi.org/10.5281/zenodo.4999446

**Software and algorithms**		

GROMACS	Abraham et al., 2015	www.gromacs.org
PyMOL	maintained and distributed by www.schrodinger.com	https://pymol.org/
Modeller	Fiser and Sali 2003	https://salilab.org/modeller/
Martini 2.2	de Jong et al., 2013	http://cgmartini.nl/
Python 3.4	Open source software	https://www.python.org/download/releases/3.4.0/
SciPy v0.19.1	Open source software	https://www.scipy.org
VMD 1.9.2	Humphrey et al., 1996	http://www.ks.uiuc.edu/Research/vmd/
martinize.py	de Jong et al., 2013	https://github.com/cgmartini/martinize.py
insane.py	Wassenaar et al., 2015	http://www.cgmartini.nl/images/tools/insane/insane.py
backward.py	Wassenaar et al., 2014	http://www.cgmartini.nl/index.php/tools2/resolution-transformation
PyLipID	https://doi.org/10.5281/zenodo.4999404	https://doi.org/10.5281/zenodo.4999404
pyEMMA	Scherer et al., 2015	http://emma-project.org/latest/

### Resource availability

#### Lead contact

Further information and requests for resources should be directed to and will be fulfilled by the Lead Contact, Mark Sansom (mark.sansom@bioch.ox.ac.uk).

#### Materials availability

No unique reagents or materials were generated in this study.

#### Data and code availability

This study did not generate new software. The simulation trajectory datasets supporting the current study have not been deposited as a public repository for MD simulation data does not yet exist. Coordinates of the models generated by this study are available from the corresponding author on request. A repository of the 9-copy oligomer atomistic models can be found at http://doi.org/10.5281/zenodo.4300676. The PyLipID analysis software can be found at https://doi.org/10.5281/zenodo.4999404. Details of lipid interaction data can be found at https://doi.org/10.5281/zenodo.4999446.

Any additional information required to reanalyze the data reported in this paper is available from the lead contact upon request.

### Experimental model and subject details

No experimental models were used. The experimental data for the MD simulations consisted of the protein coordinate set (i.e. PDB files) as detailed in the Key Resources Table.

### Method details

#### MD simulation set-up

Simulations used the MARTINI coarse-grained forcefield (de Jong et al., 2013) as previously applied in simulations of monomeric A2aR (Song et al., 2019). Nine or sixteen copies of the receptor in a given conformational state were individually rotated through a random angle at the center of a box of size of 45 × 45 × 25 nm^3^ and then randomly translated in the *xy* plane. Both randomization processes were carried out by the random module of NumPy. An even distribution was made sure in the starting configurations. The resultant configuration was embedded in a membrane bilayer with the specified complex lipid composition (see Table 1) using the *insane.py* script (Wassenaar et al., 2015). Electrolyte solution corresponding to ∼0.15 M NaCl was added and additional sodium ions were added to neutralise the system. All the simulations were performed using GROMACS 5.1(Abraham et al., 2015). The CG simulations parameters were taken from (Song et al., 2019). A summary of simulations performed is provided in Table 1, amounting to a total of ∼2.6 ms of simulation data collected. Visualization used PyMol and VMD (Humphrey et al., 1996).

#### Characterisation of oligomeric configurations

The minimum distance between each pair of proteins was monitored as a function of time (Figure S1), from which the density of such distance distribution was estimated. The density estimation showed a first peak at ∼0.55 nm and a first trough at ∼0.7 nm. We found that a cut-off of 0.75 nm could best discriminate effective associations. Using this cut-off and hierarchical clustering, the number of oligomers of different orders were counted and each copy of the receptor was assigned to those identified oligomers separately for each frame. The identified oligomeric assemblies with the same oligomeric order were grouped together into an ‘oligomer pool’, and characterised, *i.e.* clustered, within each pool if the order is lower than 6. Since calculation of root-mean-square deviation (RMSD) based on structural coordinates is sensitive to the order of the receptor indexing, we set out to assign an order to the receptor copies in the oligomers. We took a reference structure randomly from each oligomer pool and calculated the coordinate RMSDs for the rest of the structures in that pool, testing all possible orders of the receptor copies. The order giving the lowest RMSD was assigned to the structure. The oligomeric structures in each oligomer pool were then clustered using the KMeans method based on their reordered coordinates. The number of clusters were manually tested for each oligomer pool to ensure a homogeneous distribution of structures in each cluster. These clusters were individually labeled as *A.b* where *A* was the oligomeric order and *b* the cluster identifier in their oligomer pool. To assist structural inspection, the coarse-grained oligomer models were converted back to atomistic models using CHARMM 36 force field and the *backward.py* script (Wassenaar et al., 2014) provided by MARTINI website. A repository of the 9-copy oligomer atomistic models can be found at http://doi.org/10.5281/zenodo.4300676.

#### Calculation of oligomer residence time

For every oligomer cluster, the durations of their continuous appearances in the simulations were recorded. Of note, visual inspection of trajectories revealed that protein-protein interactions can have quick flickering in contact distance that does not lead to 'real' dissociations in MARTINI coarse-grained force field. Hence, we treated the protein-protein associations as 'being continuous' if their inter-protomer contacts broke for a time period shorter than 100 ns. The oligomer residence time was calculated from the normalised survival time-correlation function σ(t):

(Equation 1) $$\sigma \left(t\right)=\frac{1}{N_{j}}\frac{1}{T-t}\overset{N_{j}}{\underset{j=1}{\sum }}\overset{T}{\underset{\nu =0}{\sum }}{\tilde{n}}_{j}\left(\nu ,v+t\right)$$

where T is the total simulation time, *N*_*j*_ the number of continuous appearances collected from simulations, and ${\tilde{n}}_{j}\left(\nu ,v+\text{Δ}t\right)$ is a function that takes the value 1 if the oligomer appeared for a continuous duration of Δt after forming the contact at time v, and takes the value 0 if otherwise. Biexponentials $y=Ae^{-k_{1}\text{Δ}t}+Be^{-k_{2}\text{Δ}t}$ were used to fit the survival curve (Figure 3A). The smaller k was regarded as oligomer $k_{off}$ and the oligomer residence time was calculated as $\frac{1}{koff}$.

#### Characterisation of lipid interactions

The lipid binding sites, and binding kinetics were calculated using PyLipID (https://doi.org/10.5281/zenodo.4999404). For lipid interactions with a given residue, a continuous lipid contact was defined using a dual cut-off scheme such that the interaction started when lipid head group moved to a given residue closer than 0.55 nm and ended when the head group moved farther than 1.0 nm. The residue-wise lipid residence time was calculated from the durations of these continuous contacts using function (1) where *N*_*j*_ the number of continuous lipid contacts with the residue collected from simulations. Lipid binding sites were derived from the lipid contact information using graph theory and community analysis. Graphs were constructed using residues as nodes and the frequency with which a given pair of residues interacted with the same lipid molecule as edges. The best partition function of the community library (https://github.com/taynaud/python-louvain) was used to detect community structures. Each calculated community was considered a binding site. A continuous lipid contact with a given binding site starts as the lipid head group moved closer than 0.55 nm to any of the residues in the binding site and ends as the head group moved farther than 1.0 nm. Similar to the residue-wise residence time, the lipid residence time of a given binding site was calculated from the durations of these continuous binding site contacts using function (1) where *N*_*j*_ the number of continuous lipid contacts with the binding site collected from simulations. The analysis data of PIP_2_ and DOPS interactions with A2aR can be found at https://doi.org/10.5281/zenodo.4999446.

#### MSM and validation

Markov state models were used to understand A2aR oligomerisation network. Since the oligomerisation process of a receptor is dependent on the state of the rest of the membrane, we built MSMs using the oligomerisation state of the membrane. The number of oligomers of various orders were counted at every simulation frame to represent the oligomerisation state of the membrane, which were labeled as *A*^*a*^*-B*^*b*^*-C*^*c*^ in which *A, B, C* denote the oligomeric orders and *a, b, c* represent the number of oligomers of the denoting order in the membrane. Note that we did not consider specific oligomer structures in our MSMs so as to prevent a degree of freedom that would have prevented convergence of an MSM. The transitions between these oligomerisation states were counted and the transition matrix *P*_*ij*_ which mapped out the probability of transitioning from state *I* at time *t* to state *j* at time *t + τ* were estimated using Maximum likelihood estimation (Prinz et al., 2011). The transition matrix was calculated using pyEMMA (Scherer et al., 2015). The Markovian behavior of the process can be checked by plotting the implied timescales (*k*) from eigenvalues *μ* of the transition matrix *P* at various lag times *τ* based on the relationship *k = - τ/ln(μ)*. The smallest *τ,* at which the implied timescales started to converge to an unchanged rate, was taken to build the MSM. τ=5μs was used for both 9-copy systems and 9-copy NoPIP_2_ systems. The mean first passage time (mfpt) between the macrostates was calculated as described in (Singhal and Pande, 2005) via the functionality in PyEMMA. The lifetime of an oligomerisation state *S*_*i*_ was calculated as mfpt(*i,j*), wherej∈Θand j≠i.

### Quantification and statistical analysis

Statistical analysis details can be found in the relevant sections of the STAR Methods.

### Additional resources

No additional resources were generated by this study.

## Supplemental information

Document S1. Figures S1–S8 and Tables S1–S4

Document S2. Article plus supplemental information
