# Supplementary material for: Modulation of adenosine A2a receptor oligomerization by receptor activation and PIP2 interactions
Source: Structure. 2021 Nov 4;29(11):1312–1325.e3. doi: 10.1016/j.str.2021.06.015 (PMC8581623; doi:10.1016/j.str.2021.06.015)
Supplement: Document S1. Figures S1–S8 and Tables S1–S4 [file mmc1.pdf]

Structure, Volume *29*

Supplemental Information

Modulation of adenosine A2a receptor oligomerization by receptor activation and PIP_2 interactions

Wanling Song, Anna L. Duncan, and Mark S.P. Sansom

Supplemental Information for:

Modulation of Adenosine A2a Receptor Oligomerisation by Receptor Activation and PIP_2 Interactions

Wanling Song, Anna L. Duncan, & Mark S.P. Sansom

Department of Biochemistry, University of Oxford, UK

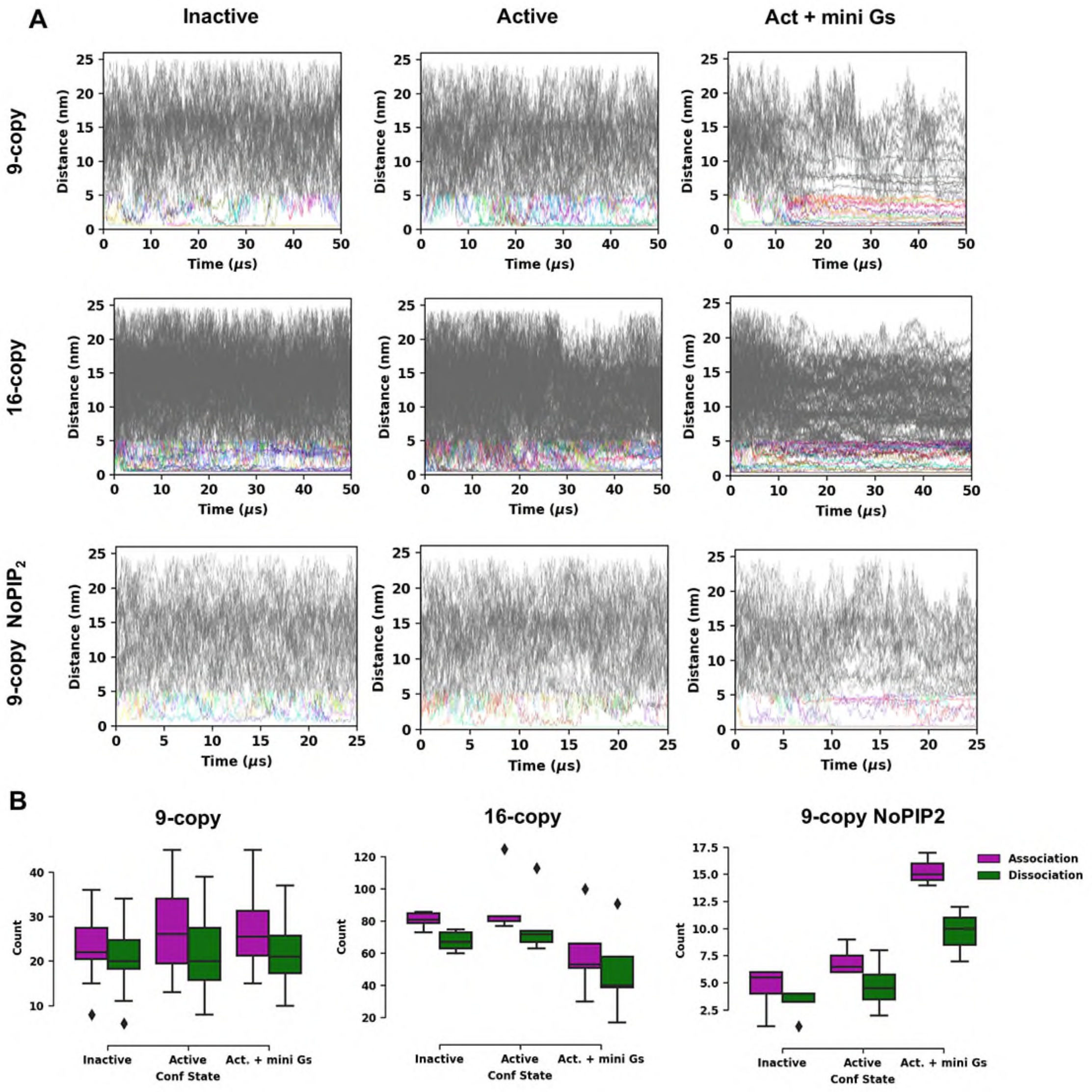

SI Figure S1. Associations and dissociations sampled in simulations. (A) Pair-wise minimum distance vs. time is shown for one example of a trajectory from each simulation ensemble. The minimum distance between each pair of proteins in the selected trajectory is plotted as a function of time. Distances below 5 nm are differentiated using different colours and coloured in grey when above 5 nm. (B) The number of associations (defined as a pair of proteins coming closer than 0.75 nm) and dissociations (defined as a pair of proteins separating to further than 0.75 nm) sampled in each trajectory of the simulation ensembles. Standard box plot settings were used, *i.e.* the boxes covering the interquartile range (IQR) between the lower and upper quartiles and whiskers showing a distance of 1.5 IQR above the upper quartile. Related to Figure 1.

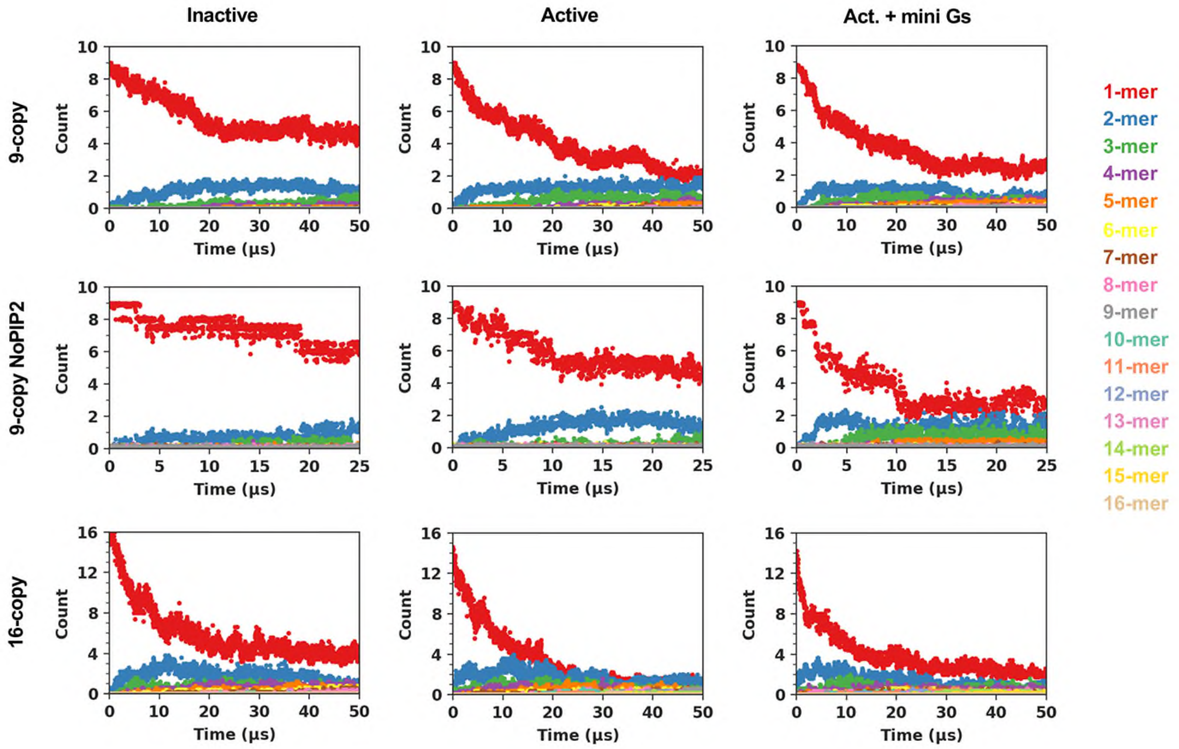

SI Figure S2. Time course of oligomer formation. The number of oligomers of various orders were monitored along the time course of simulation trajectories. The averages of data from trajectories of the same system setup were plotted as a function of time. Related to Figure 1.

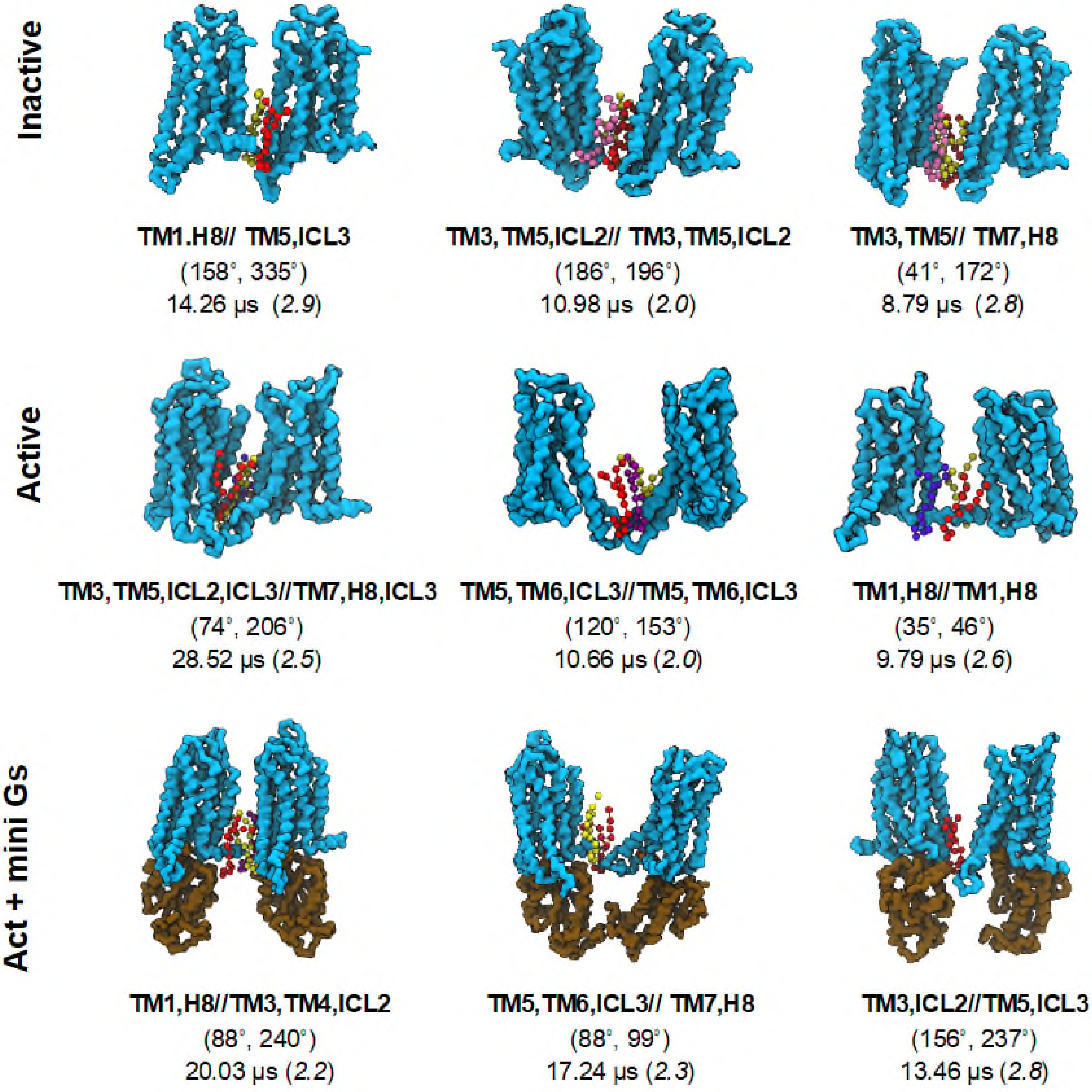

SI Figure S3. Stable dimeric configurations from 9-copy simulations. The 3 dimeric configurations with the longest residence time were shown for each conformational state. The backbone beads of the receptor are shown in cyan surface and those of mini Gs in brown surface. The PIP_2 molecules at the association interfaces are shown in ball and sticks with different colours. Below each of the structures are the association interfaces in bold, the average values of the binding angles, and the dimer residence time which is followed by the cluster id label in brackets. A table of dimeric configuration profiles can be found in SI Table S1. The atomistic models of the dimeric configurations can be downloaded at http://doi.org/10.5281/zenodo.4300676. Related to Figure 5.

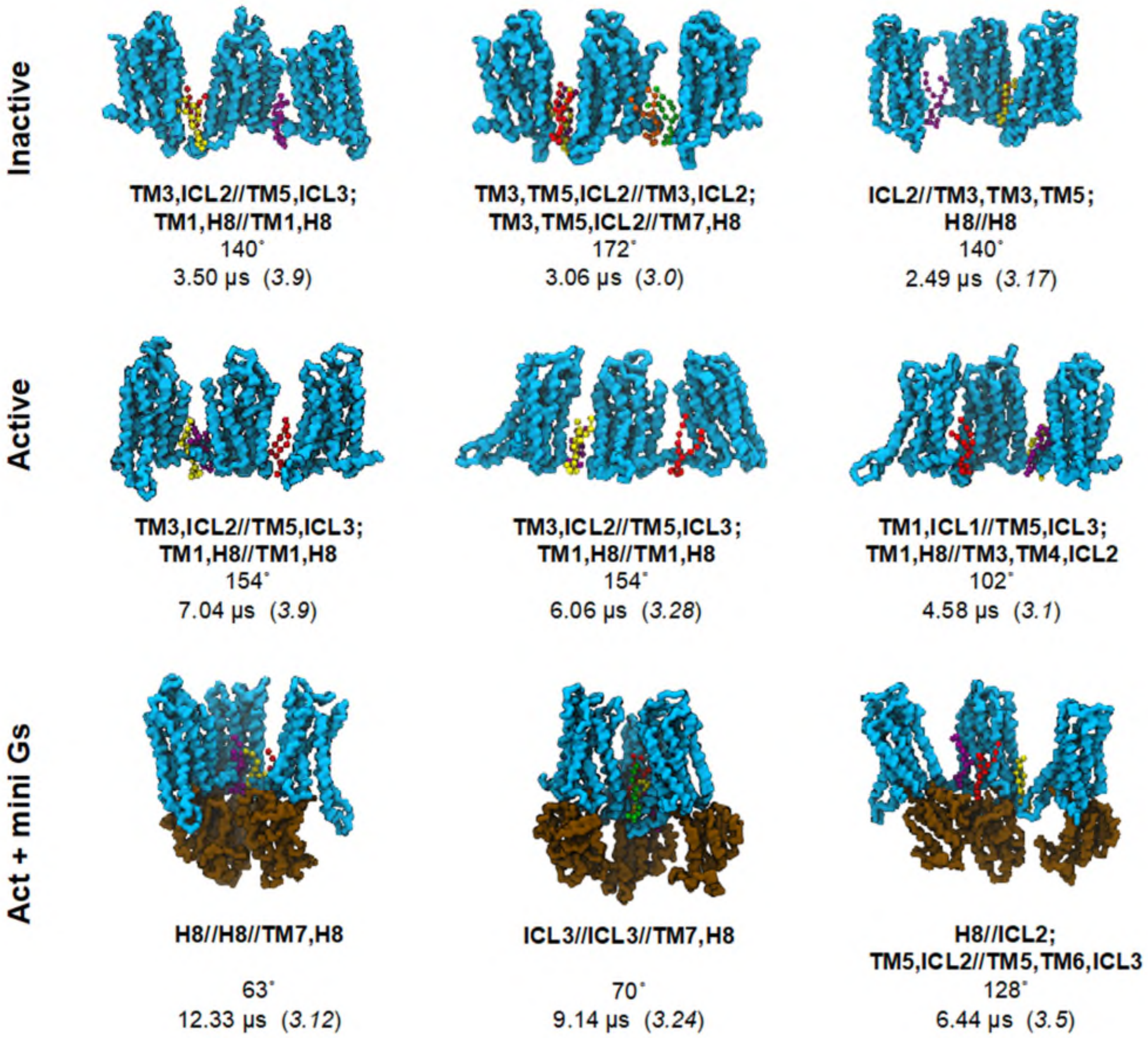

SI Figure S4. Stable trimer configurations from 9-copy simulations. The 3 trimeric configurations with the longest residence time were shown for each conformational state. The backbone beads of the receptor are shown in cyan surface and those of mini Gs in brown surface. The PIP_2 molecules at the association interfaces are shown in ball and sticks with different colours. Below each of the structures are the association interfaces in bold, the average values of the binding angles, and the trimer residence time which is followed by the cluster id label in brackets. A table of trimeric configuration profiles together with the atomistic models of the trimeric configurations can be downloaded at http://doi.org/10.5281/zenodo.4300676. Related to Figure 5.

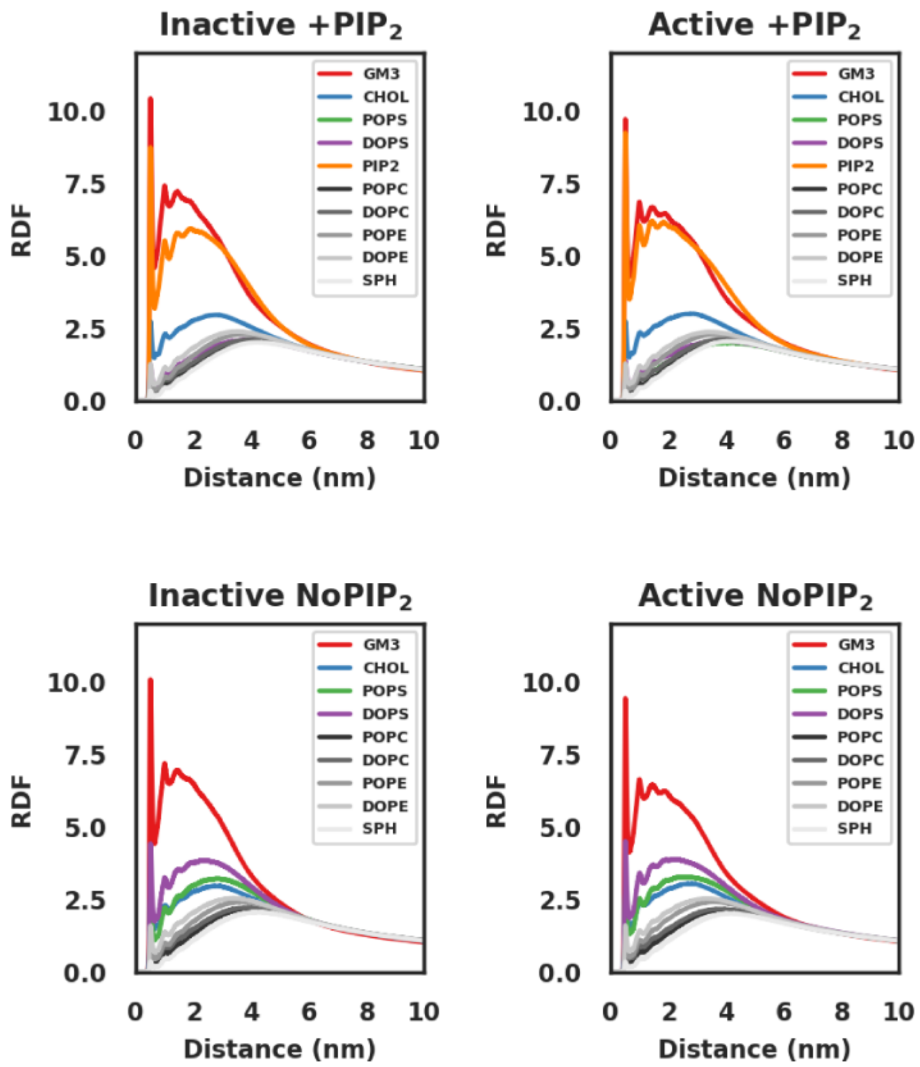

SI Figure S5. Lipid Radial distribution function around the receptor in the 9-copy simulations (+PIP_2) and the 9-copy NoPIP_2 simulations. Related to Figure 6.

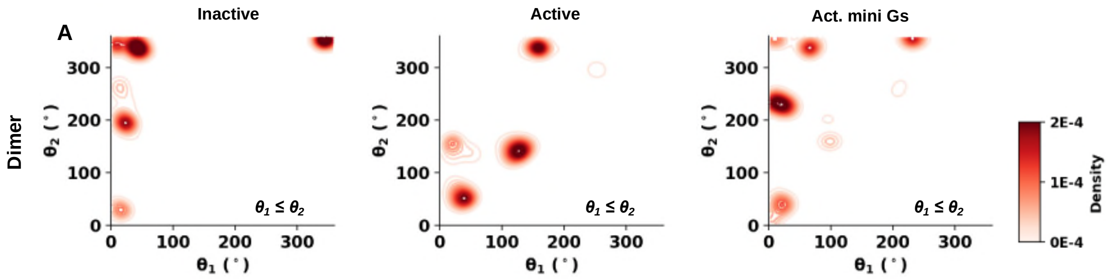

SI Figure S6. Dimer binding angles in 9-copy NoPIP2 simulations. The binding angle is defined as the angle between dimer binding interface and the principal axis that is parallel to H8, as illustrated in Fig 3B. Related to Figure 6.

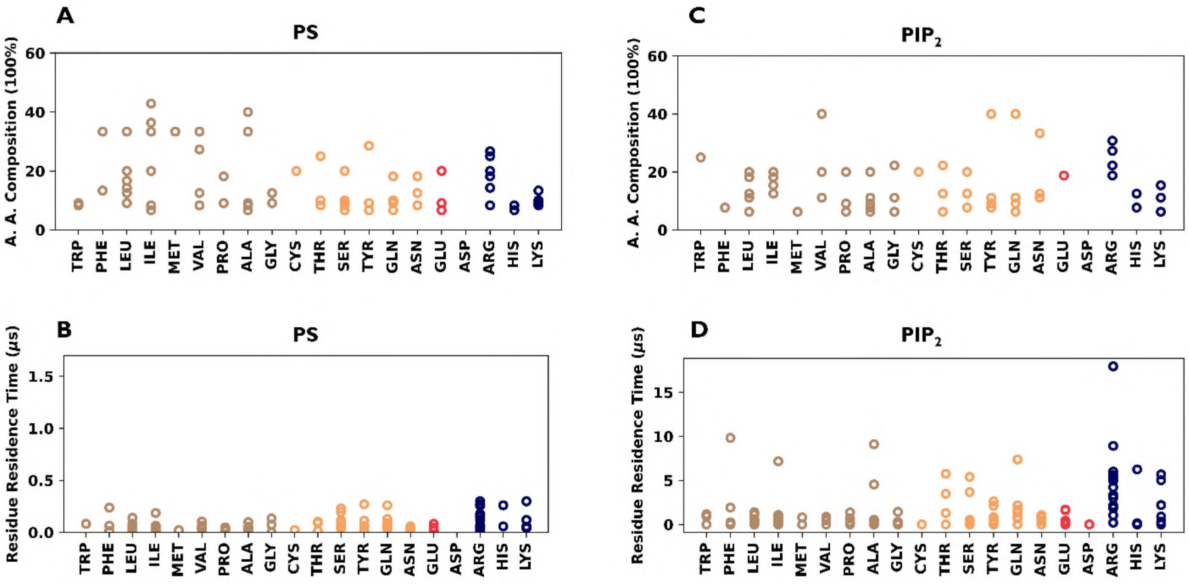

SI Figure S7. Analysis of PS and PIP_2 binding sites. The amino acid (a.a.) compositions of PS (**A**) and PIP_2 (**C**) binding sites were calculated as $\frac{Num(selected\ a.a)}{Num(binding\ site\ residues\ )} \times 100\%$. The residue residence times of PS (**A**) and PIP_2 (**C**) binding sites showed the lipid residence times for the binding site residues. Data from all binding site residues were plotted and coloured based on their chemical properties: hydrophobic residues in maroon, polar residues in orange, acidic residues in red, and basic residues in blue. Related to Figure 6.

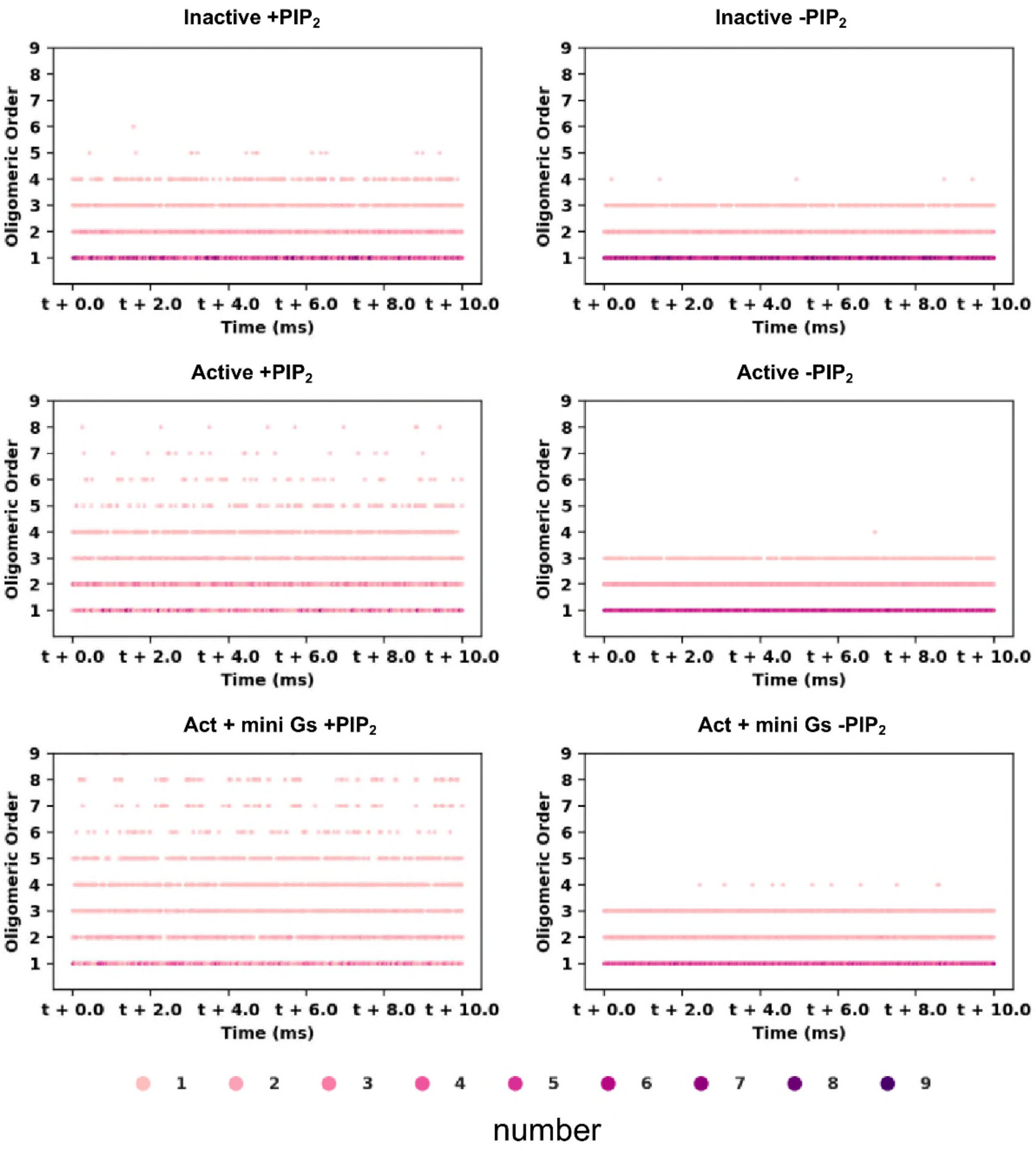

SI Figure S8. Oligomerisation kinetics in the 9-copy systems. A 10 ms trajectory was generated for each of the conformational state in 9-copy (+PIP_2) and 9-copy NoPIP2 (-PIP_2) systems using Monte Carlo algorithm and MSM transition probability. Related to Figure 7.

SI Table S1 Dimer configurations in 9-copy systems. The table is ranked by oligomer residence time and grouped by receptor conformational states. (θ_1, θ_2) is the orientation angles for dimers that describes the relative position of the dimer interface to the principal axis of that monomer that is parallel to H8. A full list of the profiles of oligomer quaternary structures in 9-copy systems can be downloaded at http://doi.org/10.5281/zenodo.4300676 along with the atomistic models of the oligomer structures. Related to Figure 5.

Conf State	Cluster id	θ_1 mean	θ_1 standard deviation	θ_2 mean	θ_2 standard deviation	Count	Pop. within Oligomeric Order (100%)	Pop. within Conf. State (100%)	Residence Time (μs)
Inactive	2.9	158.1	14.055	335.68	16.69	1760	6.18	4.8	14.268
Inactive	2.0	186.57	13.608	196.55	18.33	4994	17.534	13.62	10.98
Inactive	2.8	41.26	11.348	172.92	18.8	9627	33.801	26.25	8.789
Inactive	2.2	31.539	9.326	30.041	11.01	770	2.704	2.1	6.668
Inactive	2.1	45.46	88.124	179.3	151.4	3997	14.034	10.9	5.987
Inactive	2.6	42.082	58.813	242.7	31.6	1270	4.459	3.463	2.954
Inactive	2.3	96.869	32.643	164.29	22.83	2690	9.445	7.336	2.43
Inactive	2.10	28.832	30.525	127.33	61.27	388	1.362	1.058	2.131
Inactive	2.4	34.068	27.016	214.89	21.62	1383	4.856	3.771	1.446
Inactive	2.5	164.5	15.639	262.3	15.74	554	1.945	1.511	1.346
Inactive	2.7	60.735	12.125	119.58	11.48	1048	3.68	2.858	0.831
Active	2.5	73.04	10.21	206.7	14.67	7588	24.723	15.59	28.523
Active	2.0	120.37	19.451	153.37	22.53	7238	23.583	14.87	10.66
Active	2.6	35.107	12.243	46.137	38.7	2867	9.341	5.891	9.796

Active	2.9	96.956	9.334	114.78	10.62	3065	9.986	6.298	4.298
Active	2.7	216.1	15.216	249.09	15.18	738	2.405	1.517	3.962
Active	2.4	43.788	10.18	259.36	11.67	1563	5.093	3.212	3.619
Active	2.11	119.32	58.137	293.1	72.73	539	1.756	1.108	3.322
Active	2.8	150.86	18.935	254.34	27.11	2276	7.416	4.677	3.232
Active	2.1	67.279	20.193	138	20.02	3912	12.746	8.039	2.904
Active	2.3	39.024	39.043	162.26	76.17	565	1.841	1.161	2.18
Active	2.2	218.42	8.567	322.2	5.257	185	0.603	0.38	1.818
Active	2.10	252.32	10.773	289.26	9.706	156	0.508	0.321	1.286
Act + mini Gs	2.2	33.593	37.248	246.08	21.18	2706	11.642	6.001	20.038
Act + mini Gs	2.3	88.643	10.232	99.881	6.386	2469	10.622	5.475	17.246
Act + mini Gs	2.8	156.66	29.031	237.3	21.21	2840	12.218	6.298	13.469
Act + mini Gs	2.4	49.66	42.756	177.28	60.4	8032	34.555	17.81	12.181
Act + mini Gs	2.6	27.825	6.341	41.415	25.77	1167	5.021	2.588	11.188
Act + mini Gs	2.0	57.736	11.759	225.47	103.2	747	3.214	1.657	9.264
Act + mini Gs	2.1	99.162	9.353	322.22	22.61	1163	5.003	2.579	7.631
Act + mini Gs	2.7	133.43	15.946	161.57	18.78	1793	7.714	3.976	5.22
Act + mini Gs	2.9	192.1	22.976	268	11.22	834	3.588	1.85	4.113
Act + mini Gs	2.5	89.807	55.375	257.91	102.5	1433	6.165	3.178	3.758

SI Table S2 Dimer configurations in 9-copy NoPIP2 systems. The table is ranked by oligomer residence time and grouped by receptor conformational states. (θ_1, θ_2) is the orientation angles for dimers that describes the relative position of the dimer interface to the principal axis of that monomer that is parallel to H8. Related to Figure 5.

Conf State	Cluster ID	θ_1 mean	θ_1 standard deviation	θ_2 mean	θ_2 standard deviation	Count	Pop. within Oligomeric Order (100%)	Pop. within Conf. State (100%)	Residence Time (μs)
Inactive	2.1	122.567	143.739	304.582	104.287	2444	70.636	62.554	4.129
Inactive	2.3	30.216	36.146	198.985	28.552	665	19.22	17.021	2.857
Inactive	2.2	41.046	69.758	261.902	30.512	244	7.052	6.245	2.332
Inactive	2.4	143.189	66.26	279.784	88.525	28	0.809	0.717	0.86
Inactive	2.0	195.38	22.889	239.899	19.596	73	2.11	1.868	0.515
Inactive	2.5	145.171	10.296	167.204	14.867	6	0.173	0.154	0.047
Active	2.2	115.828	63.94	279.229	85.747	2022	30.306	27.634	5.82
Active	2.0	36.138	9.478	56.205	17.005	1689	25.315	23.083	4.097
Active	2.5	124.762	11.296	144.383	13.628	2268	33.993	30.996	5.525
Active	2.3	46.414	12.767	137.728	11.167	304	4.556	4.155	2.235
Active	2.4	252.179	8.748	295.594	8.506	194	2.908	2.651	1.886
Active	2.1	52.152	60.229	254.401	30.742	156	2.338	2.132	1.629
Active	2.6	213.984	17.362	247.049	20.423	39	0.585	0.533	0.223
Act + mini Gs	2.5	77.782	96.107	265.205	55.63	3052	47.755	28.42	12.108

Act + mini Gs	2.2	59.922	18.823	296.279	104.887	1001	15.663	9.321	8.221
Act + mini Gs	2.6	21.064	28.705	83.135	114.278	1109	17.353	10.327	5.331
Act + mini Gs	2.0	97.71	7.33	170.843	19.55	384	6.008	3.576	3.891
Act + mini Gs	2.3	13.463	4.011	348.238	35.031	186	2.91	1.732	2.931
Act + mini Gs	2.4	43.219	107.621	262.728	153.524	327	5.117	3.045	2.194
Act + mini Gs	2.1	221.969	21.807	264.823	13.759	289	4.522	2.691	1.133
Act + mini Gs	2.7	28.605	26.413	119.852	104.548	24	0.376	0.223	1.081
Act + mini Gs	2.8	209.959	7.539	225.212	6.364	19	0.297	0.177	0.3

SI Table S3. Lifetimes of oligomerisation states calculated from MSMs of 9-copy systems calculated. The MSMs were built with a lagtime of 5 µs, thus lifetimes of shorter than 5 µs cannot be resolved. Related to Figure 7.

Oligomerisation State	Inactive (µs)	Active (µs)	Active + mini Gs (µs)
1^9	8.23	6.13	5.17
1^7-2^1	8.27	7.70	5.84
1^5-2^2	7.69	6.87	7.29
1^3-2^3	8.70	7.01	7.23
1^1-2^4	5.00	6.38	5.00
1^6-3^1	5.88	6.26	7.42
1^4-2^1-3^1	7.60	6.86	7.64
1^2-2^2-3^1	7.15	8.52	7.52
1^1-2^1-3^2	5.47	8.39	5.26
1^3-3^2	5.47	7.50	5.98
2^3-3^1	5.00	5.00	5.05
1^1-2^2-4^1	5.00	5.95	8.90
1^3-2^1-4^1	5.33	6.17	8.56
1^5-4^1	5.86	5.17	5.36
1^2-3^1-4^1	5.00	7.29	14.49
1^1-4^2	5.00	5.00	5.00
2^1-3^1-4^1	5.00	5.00	5.34
1^2-2^1-5^1	5.00	8.35	7.79
1^4-5^1	5.32	6.79	14.62

1^{1}-2^{1}-6^{1}	5.00	5.00	5.10
1^{3}-6^{1}	5.00	5.09	5.21
2^{2}-5^{1}		5.00	7.45
1^{1}-3^{1}-5^{1}		8.22	6.85
1^{2}-7^{1}		5.00	5.53
2^{1}-7^{1}		5.00	5.00
1^{1}-8^{1}		5.14	9.73
4^{1}-5^{1}			6.25
3^{1}-6^{1}			5.00
9^{1}			5.00

SI Table S4. Lifetimes of oligomerisation states calculated from MSMs of 9-copy NoPIP$_2$ systems calculated. The MSMs were built with a lagtime of 5 μs, thus lifetimes of shorter than 5 μs cannot be resolved. Related to Figure 7.

Oligomerisation State	Inactive	Active	Act. + mini Gs
1^9	11.47	5.16	
1^7-2^1	14.65	7.13	5.27
1^5-2^2	12.27	11.09	5.87
1^4-2^1-3^1	5.56	5.77	8.88
1^6-3^1	5.48	5.00	9.39
1^5-4^1	5.00		
1^3-2^3		5.57	5.00
1^2-2^2-3^1		5.00	5.02
1^3-2^1-4^1		5.00	5.00
1^3-3^2			5.89
1^1-2^1-3^2			5.00
1^2-3^1-4^1			5.00
